# Genetic, Ecological and Morphological Divergence between Populations of the Endangered Mexican Sheartail Hummingbird (*Doricha eliza*)

**DOI:** 10.1371/journal.pone.0101870

**Published:** 2014-07-03

**Authors:** Yuyini Licona-Vera, Juan Francisco Ornelas

**Affiliations:** 1 Departamento de Biología Evolutiva, Instituto de Ecología, AC, Xalapa, Veracruz, Mexico; Instituto de Higiene e Medicina Tropical, Portugal

## Abstract

The Mexican Sheartail (*Doricha eliza*), an endangered hummingbird, is endemic to Mexico where two populations have a disjunct distribution. One population is distributed along the northern tip of the Yucatan Peninsula whereas the other is mostly restricted to central Veracruz. Despite their disjunct distribution, previous work has failed to detect morphological or behavioral differences between these populations. Here we use variation in morphology, mtDNA and nuDNA sequences to determine the degree of morphological and molecular divergence between populations, their divergence time, and historical demography. We use species distribution modeling and niche divergence tests to infer the relative roles of vicariance and dispersal in driving divergence in the genus. Our Bayesian and maximum likelihood phylogenetic analyses revealed that *Doricha eliza* populations form a monophyletic clade and support their sister relationship with *D. enicura*. We found marked genetic differentiation, with reciprocal monophyly of haplotypes and highly restricted gene flow, supporting a history of isolation over the last 120,000 years. Genetic divergence between populations is consistent with the lack of overlap in environmental space and slight morphological differences between males. Our findings indicate that the divergence of the Veracruz and Yucatan populations is best explained by a combination of a short period of isolation exacerbated by subsequent divergence in climate conditions, and that rather than vicariance, the two isolated ranges of *D. eliza* are the product of recent colonization and divergence in isolation.

## Introduction

The Mexican Sheartail Hummingbird (*Doricha eliza*) is an endemic to Mexico, and globally is a near threatened species according to the IUCN Red List [1]. It is locally endangered with population declines owing to habitat loss and degradation [2], and is thus facing risk of extinction in the wild. These hummingbirds of the monophyletic assemblage Mellisugini [3], known as bees, and were originally included in the genus *Trochilus* [4]. They have since moved into different genera (*Calliphlox*, *Calothorax*, *Myrtis*, *Thaumastura*, *Rhodopis*, and *Doricha*; [4]–[5]). Although the recent use of mitochondrial DNA sequences placed *D. eliza* in the bees group [6], additional taxa likely to be nested within the Mellisugini monophyletic assemblage, including the putative sister species, the Slender Sheartail (*D. enicura*), and multiple loci are needed to fully resolve the phylogenetic position of the Mexican Sheartail within the Mellisugini [6]–[7].

Sheartails are small hummingbirds with long, arched bills and are common in semi-open scrubby areas [8]. Males with glittering, rose-pink gorgets display rocking pendulum flights (shuttle displays) to females, along with high climbs and steep dives (**Figure S1** and **Video S1**; personal observation, [8]–[11]). The breeding range of the Mexican Sheartail is divided into two widely separated geographical areas, one in central Veracruz and the other mainly on the northern fringe of the Yucatan Peninsula [8], [12]–[14]. In 2002, the Veracruz population was estimated at about 2500 individuals, and the Yucatan population at no more than 6000–10,000 individuals [12]. Both the Veracruz and Yucatan populations are declining, locally threatened [1], [15], and subject to different threats. The Veracruz population is facing severe habitat degradation as a result of livestock grazing, sugarcane cultivation and residential development [1], [13]–[15], while the Yucatan population is under pressure mainly from the development of its coastal dune habitat for tourism [1], [14]. To our knowledge, no information has been published that documents the evolutionary divergence of the Veracruz and Yucatan populations of the Mexican Sheartail. There are breeding and feeding records suggesting that these separated populations are allopatric all year round [8], [10], [12]–[15]. During the breeding season (March-August), the Yucatan population is exclusively found in a narrow coastal strip mainly in the ecotone between mangroves and tropical deciduous forest [9],[14], but also breeds in gardens and urban areas [1],[14]. The Veracruz population occurs in undisturbed, dry deciduous forest and heavily disturbed agricultural landscapes c. 25 km inland [1],[13]. There is no historical evidence that the two populations interbreed, and the question of when the two populations diverged is still open. Despite the distance between the Veracruz and Yucatan populations (c. 780 km) and the differences in the habitats occupied, there are no apparent morphological or behavioral differences [13]. However, geographic distance as a driver of the divergence between the two populations of Mexican Sheartail in isolation, has not been investigated. This question is particularly important because each population is facing different threats and in a different environment, requiring locally adapted conservation schemes.

In this study, we ask the following questions: (1) what is the phylogenetic position of *Doricha eliza* within the monophyletic assemblage of Mellisugini? (2) What is the level of genetic differentiation between the Veracruz and Yucatan populations? (3) Are disjunct populations currently connected by gene flow? (4) When did the Veracruz and Yucatan populations split? And (5) was the divergence between the two disjunct populations caused by vicariance or dispersal? To answer these questions, we conducted Bayesian and maximum likelihood phylogenetic analyses of mitochondrial and nuclear DNA markers and time estimates of intraspecific genetic divergence. We also used morphological data, genetic diversity and historical demographic indices, modeling ancestral distribution, and use of niche divergence tests to infer the history of the Veracruz and Yucatan populations and the relative roles of dispersal and vicariance in driving divergence in the genus.

## Materials and Methods

### Ethics Statement

We obtained the collecting permit to conduct this work from Mexico’s Secretaría de Medio Ambiente y Recursos Naturales, Instituto Nacional de Ecología, Dirección General de Vida Silvestre (permit number: INE SGPA/DGVS/07701/11) for the field study described. This collecting permit specifically allowed for the collection of tail feathers from the birds. Manipulation of birds in the field was minimal. Birds were captured with mist nets, measured, and their two outermost tail feathers were removed for genetic analyses before the birds were released. All procedures with birds were carried out in accordance with the Guidelines for the Use of Wild Birds in Research proposed by the North American Ornithological Council and the ethics of experimental procedures were revised and authorized by the Animal Care and Use Committee under the Graduate Studies Committee (Maestría en Biodiversidad y Sistemática; No. INECOL/SP/CAP/2012/103) of the Instituto de Ecología, A.C. (INECOL). While the field studies involve an endangered and protected species, no specific permits are required for field studies such as the one described here.

### Sample Collection

Feather samples were collected from a total of 25 *D. eliza* during the 2011 and 2012 breeding seasons. Ten hummingbirds were collected in central Veracruz at the following locations: Xalapa, El Lencero, Miradores and Chavarrillo. Feather samples were collected from 15 individuals of the Yucatan population at Rio Lagartos and Chicxulub (**Table S1**). We sequenced the mitochondrial nicotinamide adenine dinucleotide dehydrogenase subunit 2 gene (ND2) and the complete ATP synthase 6 and ATP synthase 8 coding region (ATPase), and the nuclear 20454 locus from tail feathers of 25 *D. eliza*, and sequenced or downloaded sequence data from GenBank for the sister species *D. enicura* and for the outgroups, the bee hummingbirds *Calothorax lucifer*, *C. pulcher*, *Selasphorus rufus*, *S. sasin*, *S. platycercus*, *S. calliope*, *Atthis heloisa*, *Archilochus colubris* and *Tilmatura dupontii*, and the emerald *Amazilia cyanocephala* (**Table S2**). We also obtained ND2 sequences from GenBank for an additional 17 species of the bee hummingbird group (*Archilochus alexandri, Calliphlox amethystina*, *C. bryantae*, *C. mitchellii, Calypte anna, C. costae, Chaetocercus bombus*, *Ch. mulsant*, *Doricha enicura*, *Eulidia yarrellii*, *Microstilbon burmeisteri*, *Myrmia micrura*, *Myrtis fanny*, *Rhodopis vesper*, *Selasphorus flammula* and *Thaumastura cora*), 11 representative taxa of the mountain gems group and 12 species of the emeralds group to be used for sequence alignment and as outgroups (**Table S2**).

### DNA Isolation, Amplification and Sequencing Protocols

Total genomic DNA was extracted using the Qiagen DNeasy blood and tissue extraction kit (Qiagen, Inc., Valencia, CA, USA), following the manufacturer’s protocol. Using polymerase chain reaction (PCR), we amplified fragments from three mitochondrial DNA (mtDNA) coding genes: ND2 (350 bp, primers pair L5216 and H5578 [16]); ATPase 6–8 (727 bp, primer pair L8929 and H9947 [17]); and 20454 (502 bp, primer pair 20454F and 20454R [18]). PCR reactions (20 µL total volume) for genes contained 0.72×buffer, 0.58 Mm of each dNTP, 0.4 µg/µL BSA, 0.04 U *Taq* polymerase (Promega, Madison, WI, USA), 4.0 mM MgCl_2_, and 0.29 µM of each primer. PCR reactions were performed in a 2720 thermal cycler (Applied Biosystems, Carlsbad, CA, USA) or in an Eppendorf Mastercycler thermal cycler (Eppendorf AG, Hamburg, Germany). For amplification of the ND2, cycling parameters consisted of initial denaturation at 94°C for 3 min, followed by 40 cycles at 94°C for 45 sec, annealing at 47–48°C for 45 sec, 72°C for 30 sec, and a final step at 72°C for 5 min. The protocol for amplifying ATPase 68 was an initial denaturation at 95°C for 2 min, followed by 40 cycles at 92°C for 40 sec, annealing at 47–50°C for 1 min, 73°C for 2 min, and a final step at 73°C for 3 min. Amplification of the 20454 locus included initial denaturation at 94°C for 1.30 min, followed by 40 cycles at 94°C for 30 sec, annealing at 50–52°C for 30 sec, 72°C for 45 sec, and a final step at 72°C for 10 min. PCR products were purified with QIAquick (Qiagen Inc.) and sequenced in both directions to check the validity of sequence data using the Big Dye Terminator Cycle Sequencing kit (Applied Biosystems). The products were read on a 310 automated DNA sequencer (Applied Biosystems) at the INECOL’s sequencing facility. Finally, sequences were assembled using Sequencher v4.9 (Gene Codes Corp., Ann Arbor, MI, USA) and then manually aligned using SE-AL v2.0a11 (http://tree.bio.ed.ac.uk/software/seal). All sequences are deposited under the following GenBank accession numbers: KJ710519–KJ710624 (**Table S2**).

Individual haplotypes from 20454 sequences were statistically inferred using PHASE v2.1 [19]–[20] with the following parameters: 100,000 iterations, a thinning interval of 10, and a burn-in of 1000. PHASE uses a Bayesian statistical method to determine the most probable pair of alleles or haplotypes. Heterozygous sites in nuclear sequences were identified when two different nucleotides were present at the same position in the electropherograms of both strands. Three runs were conducted to check the consistency of results obtained by examining the allele frequencies and coalescent goodness-of-fit measures estimated for each run, and only highly supported haplotype pairs (probability 0.70–0.90) were maintained.

### Phylogenetic Analyses of mtDNA and nuDNA

Phylogenetic relationships among mtDNA sequences of *D. eliza* were reconstructed using Bayesian inference (BI) and maximum likelihood (ML) methods. The BI analyses were run in MrBayes v3.12 [21] and the ML analyses in RAxML v7.4.4 [22] using the CIPRES Science Gateway [23]. Phylogenetic analyses were performed using three data sets. We first ran analyses with a ND2 data set for *Doricha eliza* and available sequences for North American and South American members of the Mellisugini clade [7] retrieved from GenBank. The second set of analyses was run with the combined mtDNA data set (ND2 and ATPase) for *D. eliza* and outgroups, and the third with the nuclear 20454 locus data set. All DNA markers used and their accession numbers are listed in **Table S2**. We used jModeltest v1.1 [24] to choose the model of molecular evolution that best fit our sequence data under the Akaike information criterion (AIC; [25]), GTR+I+G (base frequencies: A = 0.3401, C = 0.3710, G = 0. 0760, T = 0.2183; gamma distribution shape parameter = 1.1860) for ND2; GTR+I (base frequencies: A = 0.2304, C = 0.0958, G = 0.3565, T = 0.3173) for ND2+ATPase; and HKY+G (base frequencies: A = 0.2480, C = 0.2034, G = 0.2310, T = 0.3177; gamma distribution shape parameter = 0.0170) for the nuclear 20454 locus. For each data set, two parallel Markov chain Monte Carlo (MCMC) analyses were executed simultaneously, and each was run for 10 million generations, sampling every 1000 generations. A majority consensus tree was obtained (50% majority-rule), showing nodes with a posterior probability (PP) of 0.6 or more. Bayesian PP values were calculated from the sampled trees remaining after 10% burn-in samples were discarded [21] to only include trees after stationarity/convergence was reached as checked in Tracer v1.5 [31]. Nodes with PP≥95 were considered to be strongly supported [26]. The consensus tree was later visualized in FigTree v1.2.3 (http://tree.bio.ed.ac.uk/software/figtree/). The BI analyses included two additional sets of analyses using the combined data set (ND2+ATPase+20454): the first used a single model for the entire combined loci data set (the ‘unpartitioned’ analyses), and the second set employed partition-specific DNA evolution models of each gene. For each data set, two parallel Markov chain Monte Carlo (MCMC) analyses were executed simultaneously, and each was run for 50 million generations, sampling every 1000 generations. We computed Bayes factors with the harmonic means [27] to determine whether applying partition-specific models significantly improved the explanation of the data.

ML analyses were performed using default values and the same evolution models as in the Bayesian analyses. Node support for the ML tree was estimated with 1000 bootstrap replicates, and nodes were considered highly supported when bootstrap values were ≥70% [28].

### Species Tree and Divergence Time Estimation

To estimate relationships between populations of *D. eliza*, we used ND2, ATPase and 20454 sequences for all *D. eliza* samples under the multispecies coalescent method of *BEAST [29]–[30] implemented in BEAST v1.7.4 [31]. This method models the lineage sorting process between units for groups of individuals not connected by gene flow above, at, or below the species level to obtain a species tree [32]. *Doricha enicura*, *Calothorax lucifer* and *C. pulcher* were the outgroups. We employed a relaxed molecular clock model with branch rates drawn independently from a lognormal distribution and the Yule process as a tree prior under a continuous population size model. The models of molecular evolution that best fit our sequence data under the Akaike information criterion (AIC; [25]) were HKY (base frequencies: A = 0.2247, C = 0.1467, G = 0.3356, T = 0.2930) for ND2; HKY+G (base frequencies: A = 0.2325, C = 0.1003, G = 0.3430, T = 0.3242; gamma distribution shape parameter = 0.0160) for ATPase; and HKY+G (base frequencies: A = 0.2447, C = 0.1990, G = 0.2310, T = 0.3254; gamma distribution shape parameter = 0.0160) for the nuclear 20454 locus. We performed three independent runs of 10 million generations each, sampling every 1000 generations, and discarding the first 1 million generations of every replicate as burn-in. Replicate results were combined in LogCombiner v1.7.4 (http://beast.bio.ed.ac.uk/LogCombiner) and the convergence of runs was confirmed by effective sample sizes (ESS) >200 for all parameters and by visual inspection of traces within and between replicates using Tracer v1.5 [31]. The resulting posterior sample of trees was summarized in a Maximum Clade Credibility (MCC) tree using TreeAnnotator v1.7.4 (http://beast.bio.ed.ac.uk/TreeAnnotator). Nucleotide substitution models selected with jModeltest v1.1 [24] were incorporated, and we used a relaxed clock model with an uncorrelated lognormal distribution. To calibrate the tree we used the mean rates of 2.9×10^–2^ substitutions/site/lineage/million years (s/s/l/My) for ND2, 2.2×10^–2 ^s/s/l/My for ATPase, and 1×10^–3 ^s/s/l/My for 20454 based on rates obtained for Hawaiian honeycreepers [33]. We prefer the rates suggested by Lerner et al. [33] because these are likely more appropriate for the lower taxonomic level of *Doricha* species [34]–[35] than the low substitution rates obtained for major bird orders [36].

BEAST v1.7.4 [31] was used to estimate the time of the most recent common ancestor (TMRCA) of clades in *D. eliza*. We ran two individual analyses to estimate TMRCA, one with all the ND2 sequences of bee hummingbirds available from previous studies [6]–[7],[37]–[38] and retrieved from GenBank, and the second with the mtDNA sequences (ND2 and ATPase) generated in our study for *D. eliza* and the other hummingbird species listed above. All sequences used and those retrieved from GenBank are listed with their accession numbers in **Table S2**. The best-fit model of evolution, GTR+I+G for the ND2 and GTR+I for the ND2+ATPase data set, was estimated from the data sets using jModeltest and an uncorrelated lognormal relaxed model selected in BEAST as the clock model. A coalescent model assuming constant population size was used to model the tree prior. The coalescent tree prior used in these analyses appears to fit better when mixed data sets are predominantly intraspecific data [39]. To calibrate the root in both analyses, we used the divergence time between the bee and emerald hummingbird groups (normal prior, mean 13.97 Ma, SD 3.0; [6]) as a secondary calibration. To calibrate the tree, we used the average divergence time for the basal split between North and South American bee hummingbirds (normal prior, mean 6.1, SD 1.0, range of 7.74-4.45 Ma; [40]). Twenty-seven species from the bee hummingbird group, and representatives of the mountain gems (11 taxa) and emeralds (13 taxa) were included as outgroups in the analysis using the ND2 data set, and a fewer outgroups were used in the analysis using the ND2+ATPase data set separately (see Sample Collection; **Table S2**). All of the samples of *D. eliza* were used in both analyses, rather than just the unique haplotypes, to avoid overestimating evolutionary time scales [41]. For each of the analyses, we performed three independent runs of 10 million generations, sampling every 1000 steps, and discarding the first 10% of trees as burn-in. We combined the log and trees files from each independent run using LogCombiner, then viewed the combined log file in Tracer to ensure that ESS values for all priors and the posterior distribution were >200, and then annotated the trees using TreeAnnotator summarized as a maximum clade credibility tree with mean divergence times and 95% highest posterior density (HPD) intervals of age estimates, visualized in FigTree.

### Genetic Structure and Genetic Diversity

To infer genealogical relationships among haplotypes, the ND2, ATPase and phased 20454 sequence data sets were separately analyzed using the statistical parsimony algorithm, implemented in TCS v2.1 [42] with the 95% connection limit. The genetic structure of mtDNA sequence data was further explored through pairwise comparisons of *F*_ST_ values and analysis of molecular variance (AMOVA [43]). The AMOVA was run grouping individuals into two groups according to the observed divergence in the BI analysis (see Results), and using the Jukes and Cantor model, and 16,000 permutations to determine the significance of the AMOVA using Arlequin v3.1 [44]. Lastly, we calculated corrected genetic distances [45] for mtDNA data sets between populations of *D. eliza* and other species within the Mellisugini clade (*D. enicura*, *Calothorax pulcher*, *C. lucifer*) using DnaSP v5.1 [46], and assessed genetic variation within populations by calculating the haplotype diversity (*Hd*) and nucleotide diversity (*π*) using Arlequin [44].

### Historical Demography

The demographic histories of the Veracruz and Yucatan populations of *D. eliza* were inferred by means of neutrality tests and mismatch distributions carried out in Arlequin v3.1 [44]. Tajima’s *D* [47] and Fu’s *Fs* [48] were calculated to test whether populations evolved under neutrality, and mismatch distributions [49] were calculated using the sudden expansion model [50] with 1000 bootstrap replicates. The validity of the sudden expansion assumption was determined using the sum of squares differences (SSD), which is higher in stable, nonexpanding populations [51]. To validate the estimated demographic and geographic expansion tests 16,000 permutations were used in Arlequin. We also used Bayesian skyline plots (BSP; [52]) to assess changes in effective population size (*Ne*) over time in BEAST. This analysis was performed for each genetic group separately and for the two groups combined (concatenated). Concatenated analysis has been proposed to satisfy the assumption of lineages interbreeding in scenarios where divergence is recent and there is low genetic structure [53]. The time axis was scaled using the mean rates of 2.9×10^–2^ substitutions/site/lineage/million years (s/s/l/My) for ND2 and 2.2×10^–2 ^s/s/l/My for ATPase based on rates for Hawaiian honeycreepers [33].

We used the ‘isolation-with-migration’ coalescent model as implemented in the program IMa [54]–[55] to estimate the time of divergence (*t*) between the Veracruz and Yucatan populations of *D. eliza*, the effective number of migrants per generation (*m*_V to Y_ and *m*_Y to V_), and the effective population size of the ancestral (*q*_A_) and descendant populations (*q*_V_ and *q*_Y_). We used mitochondrial and nuclear phased haplotypes to produce maximum-likelihood estimates and confidence intervals for splitting times, effective population sizes, and gene flow [55]. Every locus was tested for evidence of recombination using IMgc [56]. This program removes either sites or haplotypes to obtain the longest region to pass the four-gamete test [57]. Three independent runs of 25 million generations were performed under Hasegawa-Kishino-Yano (HKY) model for mitochondrial loci and the Infinite Sites (IS) model for nuclear locus. Each run used identical conditions, but different starting seed values, and a burn-in period of 3 million steps with parameter values empirically determined in the preliminary runs to verify the convergence of independent analyses. To improve the mixing of the Markov chains (to facilitate convergence), we ran multiple heated chains and kept monitoring the autocorrelation and estimates of ESS [55]. Using estimated ESS values in IMa [55], we considered stationarity to have been reached when the ESS value for each independent run was >50. The rates of 2.9×10^–2^ substitutions/site/lineage/million years (s/s/l/My) for ND2, 2.2×10^–2 ^s/s/l/My for ATPase and 1×10^–3 ^s/s/l/My for 20454 obtained for Hawaiian honeycreepers [33] were provided in the IMa input file and the mean rates for all genes were used to estimate the effective population sizes of each genetic group. We used a 2.5 year generation time assuming that the sexual maturity of Mexican Sheartail begins approximately 2 years after hatching and assuming an annual survivorship of 0.35, as estimated for other bee hummingbirds [58]–[59], to convert the effective population size estimates. Migration rates per generation were converted to population migration rates per generation using estimates of the effective population size. The approximate average generation time (T) is calculated according to T = *a*+[*s*/(1–*s*)] [60]–[61], where *a* is the time to maturity and *s* is the adult annual survival rate. Based on this, the estimate for T was 3.04 years. To convert the time since divergence parameter of IMa to years, *t*, we divided the time parameter (*B*) by the mutation rate per year (*U*) converted to per locus rate by multiplying by the fragment length in base pairs, and calculated for the rates described above.

### Species Distribution Models

We constructed a species distribution model (SDM [62]) to predict where populations of *D. eliza* resided during the Last Glacial Maximum (LGM, 21,000-18,000 years ago) and Last Interglacial (LIG, 120,000–140,000 years ago). We assembled a data set of 121 unique records (51 for Veracruz and 76 for the Yucatan) from georeferenced museum (Atlas Aves de México, [63]) specimens obtained through http://vertnet.org and the Global Biodiversity Information Facility (GBIF, http://data.gbif.org/species/browse/taxon), and analyzed the data with the maximum entropy algorithm in MaxEnt [64]–[65]. Present climate layers (temperature and precipitation variables, BIO1–BIO19) were drawn from the WorldClim database (*c*. 1 km^2^; [66]). Using ArcView v3.2 (ESRI, Redlands, CA, USA), we first extracted GIS data from the 19 WorldClim layers at *D. eliza*’s occurrence points, and then ran a correlation analysis to eliminate correlated environmental variables using the program PAST v2.12 [67]. When the correlation coefficient was higher than 0.80 the variables were considered highly correlated, and for each pair of correlated variables we selected the one that was more temporally inclusive. After removing the highly correlated variables, six variables were used in the analysis (BIO1 [Annual Mean Temperature], BIO2 [Mean Diurnal Range], BIO3 [Isothermality], BIO4 [Temperature Seasonality], BIO12 [Annual Precipitation], and BIO14 [Precipitation of Driest Month]). MaxEnt was set to randomly use 70% of the values for training and 30% of values for testing the model. We constructed the species distribution models using MaxEnt because it provides robust performance with small sample sizes (restricted distribution) of presence only data [64]. Model performance was evaluated using the area under the receiver operating characteristic curve (AUC; [68]). The model for the present was also projected to past climate scenarios, and past climate layers were drawn from WorldClim for two LGM past climate scenarios developed by the Paleoclimate Modelling Intercomparison Project Phase II [69]: the Community Climate System Model (CCSM; [70]) and the Model for Interdisciplinary Research on Climate (MIROC; [71]), and for the LIG [72]. Both CCSM and MIROC climate models simulate climate conditions as they are calculated to have been during the LGM, with a stronger temperature decrease assumed in CCSM than in MIROC [73]. Climate suitability was displayed in ArcView v3.2. (ESRI, Redlands, CA, USA).

### Niche Divergence Tests

We employed a multivariate method [74] to test for niche divergence/conservatism. Briefly, we tested for niche divergence using climate data extracted from occurrence points and used the six uncorrelated BIO variables described above to generate ENMs, and then drew minimum convex polygons around occurrence points of each lineage using the Hawth’s Tools package in ArcMap v9 [74]. We defined the background characteristics of each group using 1000 random points inside each polygon, and then conducted a principal components analysis (PCA) using these data. The first three PC (niche) axes explained a high percentage of the variance (89%) and were thus used in further analyses. Niche divergence or conservatism was evaluated on each niche axis by comparing the observed difference between the means for each lineage on that axis to the mean difference in their background environments on the same axis [74]. A null distribution of background divergence was created by recalculating the score of background divergence over 1000 jackknife replicates with 75% replacement. Significance for rejecting the null was evaluated at the 95% level. These analyses were conducted using Stata v10 (StataCorp, College Station LP, Texas, USA).

### Morphological Variation

To examine differences in morphological variation between the mist-netted Veracruz and Yucatan adult hummingbirds used in the genetic analyses, six measures were taken using a dial calliper with a precision of 0.1 mm and a wing ruler: total body length (BL; the distance from the tip of its bill to the tip of longest tail feather); exposed culmen (EC; from the base of the bill to the tip of the upper mandible); bill width at the base (BB; by the location of the nostrils); and wing chord (WC; the distance from the carpal joint to the tip of the longest unflattened primary) for both males and females, and tail length (TL; from the uropygial gland to the tip of the longest rectrix) for females, and the length of the outermost rectrices (r5), from the base of the uropygial gland to the tip of the longest rectrix (left and right) for males. All measurements were taken by YLV. Measurements for two juvenile males from Yucatan were discarded from the analysis. To examine morphological differences between populations, for males and females we conducted a one-way non-parametric Kruskal-Wallis test using genetic group as fixed factors and morphological measures as dependent variables. These analyses were performed using SPSS v17 for Mac (SPSS, Armonk, NY, USA).

## Results

### Phylogenetic Analysis of mtDNA and nuDNA

**Figure 1:**
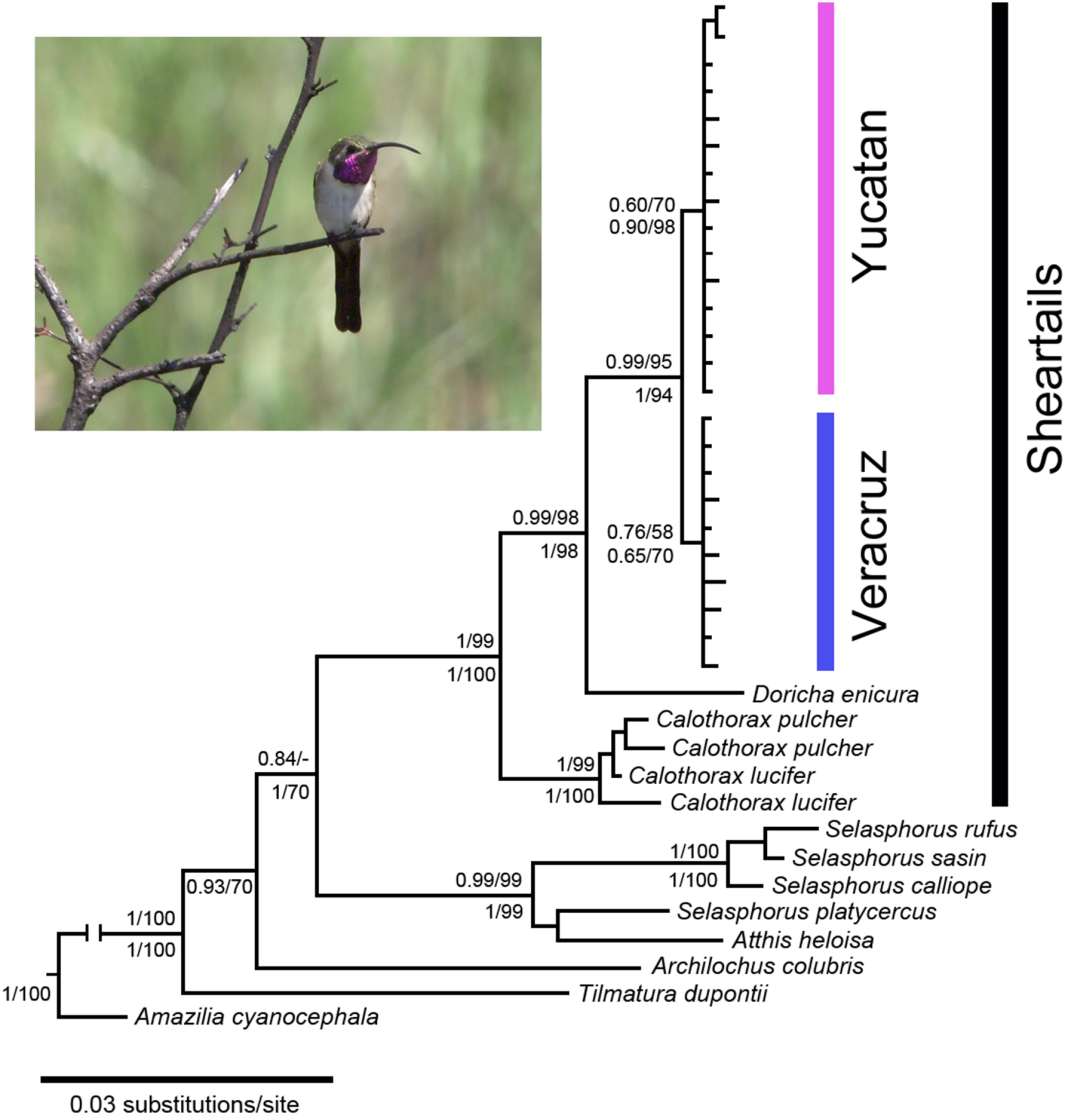
Bayesian posterior probabilities and bootstrap support for MrBayes and Maximum Likelihood analyses. Illustration of tree topology based on ND2+ATPase+20454 concatenated sequences of *Doricha eliza* and outgroups. Values above branches denote posterior probabilities (left) and bootstrap values (right) and those below branches denote the same values for phylogenetic analyses based on the ND2+ATPase data set (20454 excluded).

Interspecific phylogenetic relationships among mtDNA and nuDNA sequences of *D*. *eliza* and other species in the bee hummingbird group were reconstructed using Bayesian inference (BI) and Maximum Likelihood (ML). The BI and ML analyses yielded the same general topologies with minor differences in the position of some terminal branches. Only BI trees are shown. Both the BI and ML trees of the ND2 sequence data set confirmed that *D. eliza* and *D. enicura* form a highly supported monophyletic clade (PP = 0.95, bootstrap = 79%; **Figure S2**). A highly supported relationship between *Doricha* and *Calothorax* species was retrieved (P = 0.94, bootstrap = 75%), yet the relationships within these genera of sheartails and those between other members of the Mellisugini are not fully resolved (**Figure S2**). The level of polymorphism found in the Mellisugini of the nuclear 20454 locus was low and several haplotypes were shared among species. Most interspecific relationships in the Mellisugini were not resolved when only using 20454 (**Figure S3**). In contrast, the interspecific phylogenetic relationships among species in the Mellisugini were more fully resolved when using the combined ND2+ATPase+20454 data set (**Figure 1**). The relationship between *D. eliza* and *D. enicura* is retrieved with high support in both the BI and ML analyses (PP = 0.99, bootstrap = 98%), and monophyly of sheartails (between *Doricha* and *Calothorax* species) is also retrieved with high support (PP = 1.0, bootstrap = 99%). Individuals of *D. eliza* are retrieved as a monophyletic group (PP = 0.99, bootstrap = 95%), with a split separating the Veracruz and Yucatan populations (**Figure 1**). The BI inference using the combined data set of both mtDNA and nuDNA (ND2+ATPase+20454), with unpartitioned and partitioned DNA evolution models of each gene yielded the same relationships. The Bayes factor indicated that the BI tree obtained with the unpartitioned data was more informative (harmonic mean loglikelihood, unpartititoned = –4616.68, partitioned by each gene = –4547.25, logB10 = 69.43, 2logB10 = 138.68), and this difference was very strong (the PP values for the tree obtained with partitioned data are shown in **Figure 1**).

### Species Tree and Divergence Time Estimation

**Figure 2:**
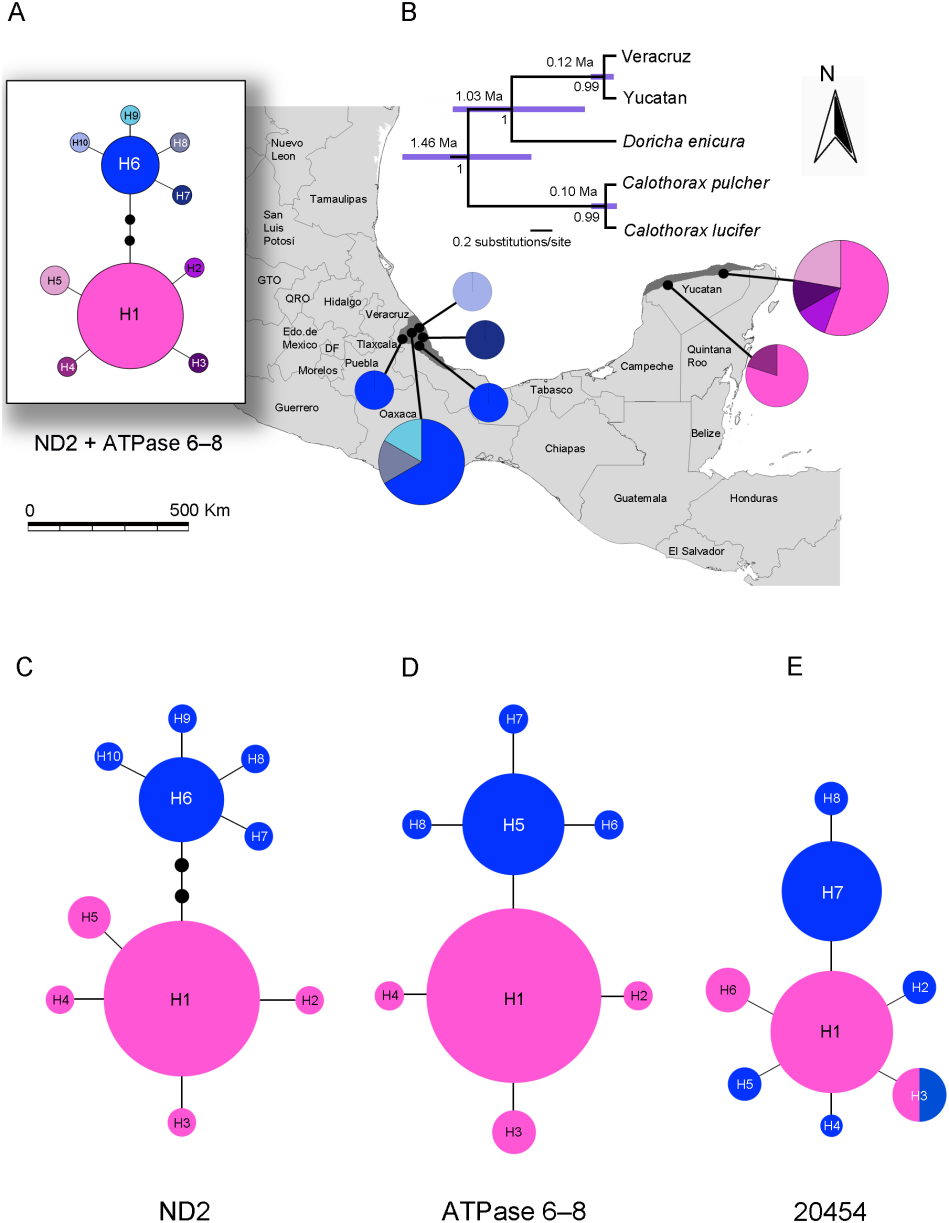
Genetic divergence of *Doricha eliza* populations in Veracruz and the Yucatan Peninsula, Mexico. (A) Haplotype network for ND2+ATPase 6–8 concatenated sequences overlaid on a relief map showing the geographical distribution of *D. eliza*. (B) Species tree and time divergence estimates (95% HPD) in years from the *BEAST analysis based on both mitochondrial (ND2+ATPase) and nuclear DNA (20454 locus). Numbers below branches denote Bayesian posterior probabilities (PP). (C) Haplotype network for ND2. (D) Haplotype network for ATPase. (E) Haplotype network for 20454. Haplotypes are represented by circles, their size proportional to their frequency in the population. Each branch represents a single nucleotide change, with additional mutations indicated by black dots along branches. The color-coding of haplotypes is the same in all figures, blue colors for Veracruz and rose-pink colors for the Yucatan.

Relationships between *D*. *eliza* populations estimated in *BEAST (**Figure 2**) strongly supported common ancestry for the Veracruz and Yucatan populations (PP = 1.0). Results from *BEAST (ND2+ATPase+20454) suggest that the divergence between the Veracruz and Yucatan clades occurred at c. 120,000 years ago (95% HPD 240,000-31,000 ka), and the divergence between *Doricha* species (TMRCA) in BEAST was estimated to be 1.03 Ma (95% HPD 1.608-0.309 Ma) and 1.46 Ma (95% HPD 2.104-0.852 Ma) between *Calothorax* species (**Figure 2**). Divergence time between *D. eliza* populations was estimated to be 541,000 years ago (95% HPD 902,000-224,000 ka, PP = 0.99) when using the ND2 data set of Mellisugini representatives and 222,000 years ago (95% HPD 352,000-107,000 ka, PP = 1.0) when using the ND2+ATPase data set. Estimates of the TMRCA for *Doricha* species and for the sheartails (*Doricha* and *Calothorax*) indicate that the splits occurred at 1.04 Ma (95% HPD 1.674-0.486 Ma, PP = 0.99) and 2.06 Ma (95% HPD 3.224-1.038 Ma, PP = 1.0) when using the ND2 data set, and at 0.7 Ma (95% HPD 1.791-0.761 Ma, PP = 1.0) and 1.21 Ma (95% HPD 1.719-0.761 Ma, PP = 1.0) when using the ND2+ATPase data set.

### Genetic Structure and Genetic Diversity

Sequencing two mtDNA markers in 25 individuals of *D. eliza* (**Tables S1 and S2**) produced 10 haplotypes for ND2, and 8 haplotypes for ATPase, resulting in 10 haplotypes for the concatenated sequence (1077 bp). Phylogenetic analysis of the ND2, ATPase, and concatenated ND2+ATPase haplotypes revealed genetic divergence between the Veracruz and Yucatan populations with no shared haplotypes (**Figure 2**). Haplogroups are connected by more than one step for the ND2, one step for the ATPase, and by four steps for the concatenated ND2+ATPase. Haplotype diversity (*h*) and nucleotide diversity (π) were moderate for both the Veracruz (*h* = 0.666±0.163, π = 0.0007±0.0006) and Yucatan (*h* = 0.561±0.143, π = 0.0006±0.005) populations. When samples were combined, overall *h* was 0.796, and overall π was 0.0020 indicating relatively high levels of genetic diversity in the Mexican Sheartail hummingbird. The AMOVA results revealed strong population structure (*F*_CT_ = 0.79, df = 1,24, *P*<0.05) when samples were grouped by geographic area. Mitochondrial divergence between the Veracruz and Yucatan populations was low (*Dxy* = 0.35%), whereas genetic divergence between populations of *D*. *eliza* and the other members of the sheartails ranged from 0.46% to 3.47% (**Table 1**).

**Table 1:** Percent genetic distances between populations corrected for intra-population polymorphism (%*Dxy*).

*X*	*Y*	*%Dxy*
Veracruz	Yucatan	0.35
Veracruz	*Doricha enicura*	2.62
Veracruz	*Calothorax lucifer*	3.43
Veracruz	*Calothorax pulcher*	3.47
Yucatan	*Doricha enicura*	2.72
Yucatan	*Calothorax lucifer*	3.33
Yucatan	*Calothorax pulcher*	3.37
*Doricha enicura*	*Calothorax lucifer*	3.48
*Doricha enicura*	*Calothorax pulcher*	3.62
*Calothorax lucifer*	*Calothorax pulcher*	0.46

Data shown for differences between the Veracruz and Yucatan populations of *D. eliza* and between species of *Doricha* and *Calothorax* based on concatenated mtDNA (1077 bp).

The sequencing of the nuDNA locus 20454 produced 8 haplotypes for the phased sequences. One haplotype was shared between the Veracruz and Yucatan populations (**Figure 2**). However, five haplotypes were found only in the Veracruz population and the other two in the Yucatan population, indicating some genetic structure in this locus.

### Historical Demography

**Figure 3:**
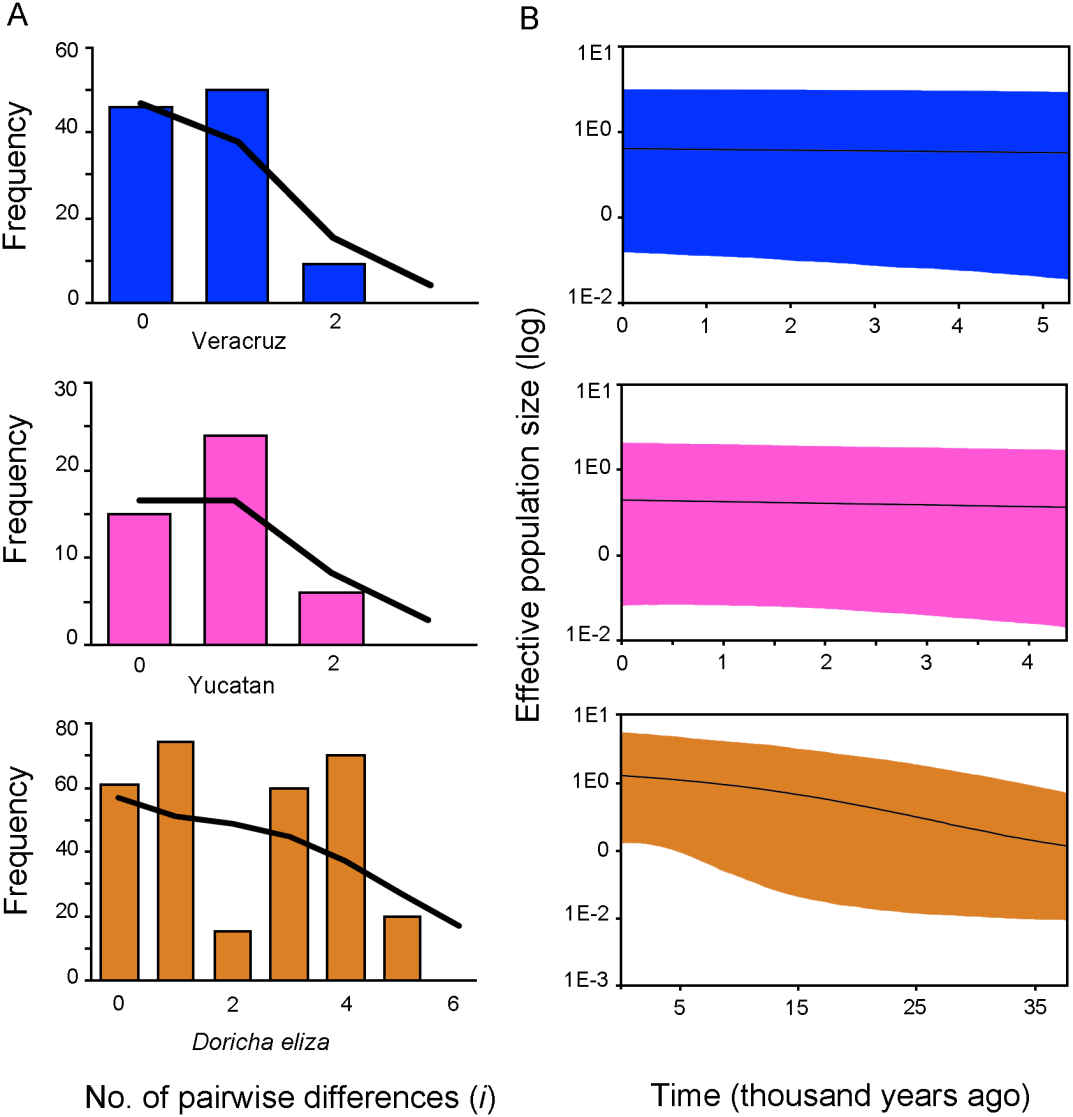
Mistmatch distributions (A) and Bayesian skyline plots (B) showing historical demographic trends of Veracruz, the Yucatan and *Doricha eliza* populations using mitochondrial sequences. Histograms correspond to observed frequencies of pairwise nucleotide differences, and lines represent the expected frequencies under a sudden expansion model. The *y* axis of the skyline plots is the product between effective population size and the generation time and the *y* axis is time in thousands of years. A mutation rate of 2.9×10^–2^ substitutions/site/lineage/million years (s/s/l/My) for ND2, 2.2×10^–2 ^s/s/l/My for ATPase, and 1×10^–3 ^s/s/l/My for 20454 based on rates obtained for Hawaiian honeycreepers [33]. Solid lines represent median estimates and shaded areas represent 95% confidence intervals. The color-coding, as in Fig. 2, is blue for Veracruz, rose-pink color for the Yucatan, and orange for all populations of *D. eliza*.

We conducted demographic analyses for the Veracruz and Yucatan populations and for all populations of *D*. *eliza* using the concatenated mtDNA data set. Neutrality tests revealed low and negative values in all cases, except that the Tajima’s *D* value for the whole population was not significant (**Table 2**). In the mismatch distribution (**Figure 3**), sudden demographic expansion (SSD values) was not rejected for all cases (**Table 2**). The BSP of *N*_e_ over time showed no evidence for population expansion; BSP for the Veracruz and Yucatan lineages were flat over time and there was an increase in population size around the LGM (c. 21,000 years ago) when the Veracruz and Yucatan populations were pooled (**Figure 3**).

**Table 2:** Results of demographic analyses of *Doricha eliza*.

Group	N	H	*D*	*Fs*	SSD
Veracruz	10	5	–1.6670*	–2.847***	0.0311*
Yucatan	15	5	–1.5181*	–2.676***	0.0170*
*Doricha eliza*	25	10	–0.8057	–3.260**	0.0337*

N = number of individuals, H = number of haplotypes, *D* = Tajima’s *D*, *Fs* = Fu’s *Fs*, SSD = differences in the sum of squares or mismatch distribution.

*P<0.05,

**P<0.01,

***P<0.001.

IMa results are summarized in **Table 3**. Results are reported as highest point estimates and 90% highest probability density (HPD). Based on the mutation rates obtained for Hawaiian honeycreepers, the ancestral population size (*N*_A_) was estimated to be 5,380 (90% HPD, 819–10,600) and the sizes of the two descendant populations were *N*_VERACRUZ_ = 1,830 (90% HPD, 1,120–2,520) and *N*_YUCATAN_ = 1,410 (90% HPD, 993–1,830). Migration rates between genetic groups (*m*_YUCATAN→VERACRUZ_ and *m*_ VERACRUZ→YUCATAN_) were 1.28 (90% HPD, 0.316–2.790) and 1.02 (90% HPD, 0.299–2.120), respectively, and the divergence time (*t*) between genetic groups was estimated to be 22,100 years ago (90% HPD, 27,000-17,400 ka).

**Table 3:** Results of isolation-with-migration model (IMa) for the splits between the Veracruz (V) and Yucatan (Y) populations of *Doricha eliza*.

	Model parameter estimates					
	q_V_	q_Y_	q_A_	t	m_V to Y_	m_Y to V_
Veracruz vs. the Yucatan						
Mean	2.213	1.712	6.538	1.917	1.155	1.192
HPD95Lo	1.360	1.206	0.994	1.505	0.465	0.495
HPD95Hi	3.056	2.206	12.883	2.341	1.823	1.911
	**Demographic parameter estimates**					
	***N*** **_V_**	***N*** **_Y_**	***N*** **_A_**	***t***	***Nm*** **_Y to V_**	***Nm*** **_V to Y_**
Veracruz vs. the Yucatan						
Mean	1,830	1,410	5,380	22,100	1.280	1.020
HPD95Lo	1,120	993	819	17,400	0.316	0.299
HPD95Hi	2,520	1,830	10,600	27,000	2.790	2.120

Model parameters indicate estimates without using the molecular rate of evolution for six parameters (IMa output values). Demographic rates represent parameters scaled to rates of molecular evolution; *q* parameters in thousands of effective population size (*Ne*), *m* in genes per generation of effective migration rate (*Nm*), *t* parameter in thousands of years.

### Species Distribution Models

**Figure 4:**
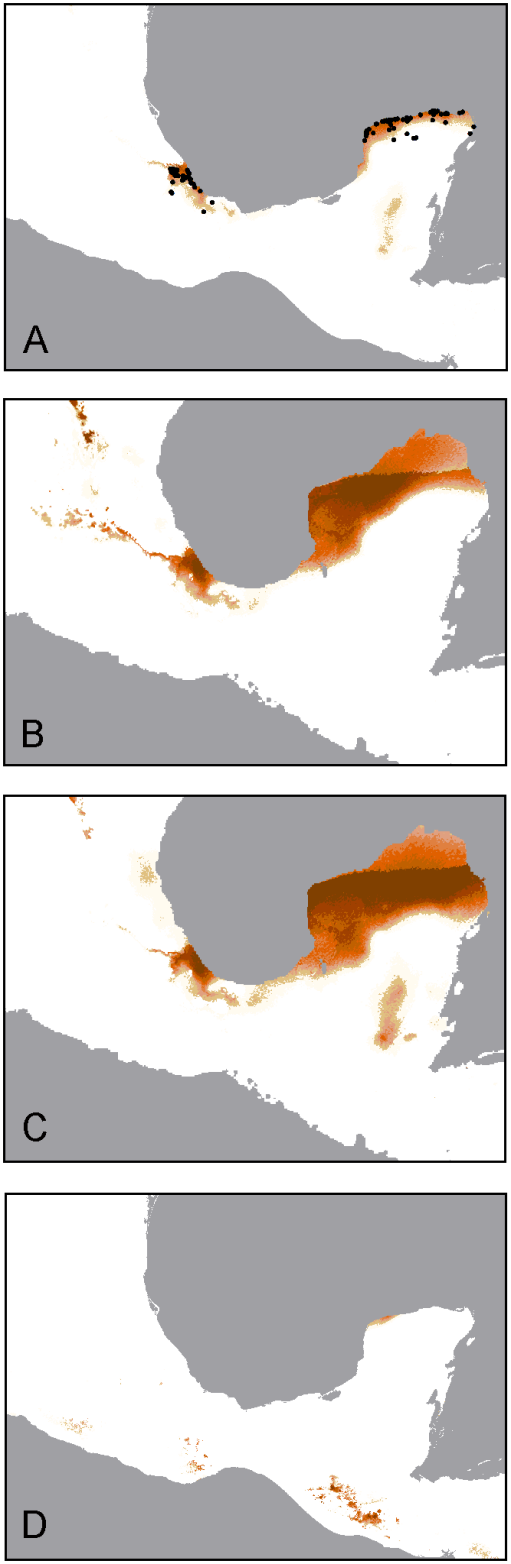
Distributional records and species distribution models for *Doricha eliza* at present (A), the Last Glacial Maximum (LGM, 21 ka) (B, CCSM model; C, MIROC model), and the Last Interglacial (LIG, 140–120 ka) (D) climate conditions. The darkest colors indicate the highest predicted probability of occurrence.

The current distribution predicted by MAXENT (**Figure 4**) closely matched the known range of *D. eliza* (**Figure 2**), and the models performed well (all AUC values >0.948). The ENM for the current climate variables using both the Veracruz and Yucatan records predicted well the distribution of the species well and over-predicted the distribution of the Veracruz population (**Figure 4**). When models were projected onto past climatic layers based on two LGM climate scenarios (MIROC and CCSM), predictions suggest that suitable habitat for both *D. eliza* populations expanded in Veracruz and the Yucatan Peninsula with a large geographical disjunction. Lastly, models projected onto LIG climatic layers revealed a different scenario to the predicted ENM for the present (**Figure 4**). Predictions suggest that there was almost no suitable habitat for *D. eliza* in Veracruz, and that potentially suitable habitat for *D. eliza* was restricted to a smaller area in the tip of the Yucatan Peninsula, small areas in the arid central valleys of Oaxaca (low probability), and Guatemala.

### Niche Divergence Tests

The PCA of environmental data that together three niche axes explained 88.7% of the variation in *D. eliza* (Veracruz and Yucatan records). The first niche axis (38.8% of variation) was associated with isothermality (BIO3) and precipitation of driest month (BIO14). The second niche axis (34.2%) was associated with annual precipitation (BIO12) and annual mean temperature (BIO1), and the third axis (15.7%) was associated with mean annual range (BIO2) and temperature seasonality (BIO4). Tests of niche divergence and conservatism on these three niche axes showed evidence for niche conservatism on niche axis 1 and niche divergence on niche axis 2 and 3 (**Table 4**).

**Table 4:** Loadings of the environmental variables for each PC axis and tests of niche divergence and conservatism.

	Niche Axes		
	PC1	PC2	PC3
BIO1 Annual Mean Temperature	–0.2471	–0.5043	0.1829
BIO2 Mean Diurnal Range	0.4634	–0.0849	0.7049
BIO3 Isothermality	0.5661	–0.3322	0.0686
BIO4 Temperature Seasonality	–0.4005	0.2751	0.6713
BIO12 Annual Precipitation	0.0082	0.6121	0.0565
BIO14 Precipitation of Driest Month	0.4932	0.4217	–0.1052
Percent variance explained	38.8	34.2	15.7
Observed differences	0.184*	**3.637** *	**0.613** *
Null distribution	(1.801–1.820)	(1.973–1.989)	(0.334–0.3353)

Observed differences in climatic niche of *Doricha eliza* lineages (Veracruz and the Yucatan) on each PC axis. Bold values indicate significant niche divergence of the differences between their environmental backgrounds compared to the middle 95th percentile of a null distribution (in parentheses).

*Significance level, *P*<0.05.

### Morphological Variation

Morphological analysis detected no significant differences in the mean values of most traits between the Veracruz and Yucatan populations (Kruskal-Wallis tests, *P*>0.05; **Table S3** and **Figures S4 and S5**), yet males from the Yucatan population had significantly smaller left outermost rectrices (r5; mean = 34.3 mm, SD = 0.05) than those from Veracruz (mean = 37.3 mm, SD = 0.11; Kruskal-Wallis test, *H* = 7.5, *P*<0.01; **Figure S4**).

## Discussion

### Mellisugini Phylogeny and the Molecular Placement of the Mexican Sheartail

A molecular phylogeny combining the available ND2 sequences to resolve the relationships within the Mellisugini (bees) clade was not available until now. Here we used a ND2 data set from four taxa assigned to the genera *Doricha* and *Calothorax*, as well as 22 samples of all other genera within Mellisugini [3] to determine the place of *D. eliza* within this phylogeny. We included our ND2 (350 bp) sequences and the available ND2 (1041 bp) sequences were downloaded from GenBank, which led to a high percentage of missing characters. Although some studies found that incomplete data could bias the ML and BI analysis [75], other studies have argued that missing data does not affect the accuracy of phylogenies in either the ML or BI analysis, and that phylogenetic accuracy is typically improved with the addition of characters even if much of the information for those characters is missing [76]–[77]. Most suspected members of the Mellisugini [3],[78], *Archilochus*, *Atthis*, *Calothorax*, *Calliphlox*, *Calypte*, *Chaetocercus*, *Doricha*, *Eulidia*, *Microstilbon*, *Myrmia*, *Myrtis*, *Rhodopis*, *Selasphorus* (incl. *Stellula*), *Thaumastura* and *Tilmatura*, are recovered within two groups (sheartails and “Selasphorus” + woodstars). A previous study [6] using mtDNA sequences confirmed the inclusion of *Doricha, Calothorax, Atthis* and *Tilmatura* in the Mellisugini as suggested by McGuire et al. [3], with *Tilmatura dupontii* as the only representative of the woodstars and sister to all other bees in that study, whereas our phylogenetic analyses place *T. dupontii* closer to South American woodstars.

According to our phylogenetic analyses of the combined data set (ND2+ATPase+20454), the Mexican Sheartail hummingbird (*D. eliza*) is strongly supported as the sister group to *D. enicura*, and together they appear as the sister to *Calothorax* species forming the group of sheartails with strong support. The relationship between sheartails and woodstars, however, received moderate support, and *Archilochus colubris* and *A. alexandri* cluster with the woodstars. In a recent study surveying Mellisugini relationships using nuclear and mtDNA sequences [78], *Archilochus* species appeared in a clade with *Calliphlox evelynae* and *Mellisuga minima* but a sister relationship between this clade and sheartails (*Calothorax lucifer* and *Doricha eliza*) was not supported. More data is necessary, including that of *Mellisuga helenae*, *Chaetocercus heliodor*, *Ch. astreans*, *Ch. berlepschi* and *Ch. jourdanii*, to verify this position and to corroborate the monophyly of woodstars. Based on the BI and ML analyses of the combined data set (ND2+ATPase+20454), we propose that *D. eliza* is sister to *D. enicura* and both form a monophyletic clade with *Calothorax* species.

### Divergence Date Estimates

An important question that is implicit to our study is how much time after isolation (or colonization) is required for genetic and morphological variation to arise in natural populations. For Mexican Sheartails, the monophyly of *D. eliza* is indicative of a single isolation or relatively recent colonization event from the Yucatan to Veracruz, perhaps in the last 120,000 years. The star-shaped haplotype network recovered in the ND2, ATPase, 20454 data sets and in the combined ND2+ATPase data set, and the lack of shared mtDNA haplotypes between the Veracruz and Yucatan populations also suggest a recent isolation or colonization followed by haplotype differentiation *in situ*. In contrast to the mtDNA pattern, one of the nuDNA locus 20454 low-frequency haplotypes is shared between populations, suggesting that the nuclear genome also became differentiated after a short history of isolation or colonization. The quasi star-shaped haplotype networks with some low frequency singletons separated from high frequency central haplotypes by a single mutational step, the moderate levels of differentiation between populations, and a mismatch distribution of pairwise differences among haplotypes indicating a sudden increase in expansion from a single population are all expected for a species that rapidly expanded from a single refugium with high levels of gene flow [51],[79]–[80]. The modeled paleodistribution suggests that suitable LGM habitat for the Mexican Sheartail would have expanded under both the MIROC and CCSM scenarios, but suitable habitat conditions were not predicted in Veracruz during the LIG. While populations may have expanded during the LGM, the disjunction persisted and, therefore, our genetic results along with those of paleodistribution modeling correspond to the hypothesis of a relatively recent colonization event from the Yucatan to Veracruz.

### Colonization

Mellisugini are a recently diverged lineage [3],[6]–[7], and are part of a radiation that includes the evolution of several species of Nearctic-Neotropical migrants [37],[81]. Despite the observed genetic differentiation between the two populations of *D. eliza*, the question of how their isolation occurred remains unanswered. It is likely that the Veracruz population represents a relatively recent colonization event, though it is difficulty to directly observe immigration events in nature [82]. Colonization has been more important than large-scale vicariance in determining the phylogenetic structure of hummingbird faunas, particularly the insular Mellisugini species assemblage of the West Indies [83], owing to their high dispersal ability, their capacity to adapt to novel environments [82]–[83], and the fact that migratory behavior can evolve rapidly in response to selection [37]. The Mellisugini are highly opportunistic generalists that, seasonally and altitudinally, cover large distances to track floral resources [5],[84]–[85]. These migratory habits confer a natural vagility and may have predisposed them to fly long distances and tolerate a wide range of ecological regimes [84]. Although migratory behavior might have increased the colonization success of Mellisugini in the West Indies and remote geographic areas with a seasonal climate, vagrancy does not appear to predict the colonization of oceanic islands or remote areas [83], and it is not known whether migratory Mellisugini species are more prone to vagrancy than sedentary hummingbird species such as the Mexican Sheartail. An alternative explanation is that ancestral colonizers arrived naturally from Yucatan to Veracruz, a direction potentially assisted by the prevailing east-to-west trade winds and hurricanes. Our estimates of historical gene flow indicating a general trend of unidirectional gene flow between populations correspond to a Yucatan-to-Veracruz direction of historical migration.

### Genetic and Morphological Differentiation between Disjunct Populations

Our results reveal moderate mtDNA divergence between the Veracruz and Yucatan populations of *D. eliza* but reciprocal monophyly of haplotypes, supporting the hypothesis of a short history of isolation. Moderate levels of haplotype and nucleotide diversity of populations suggest relatively small population sizes and founder effects. Significant genetic differentiation between populations and limited gene flow resulting from barriers to dispersal have been found for montane hummingbird species in particular, such as Speckled Hummingbird (*Adelomyia melanogenys*) [86], Wedge-tailed Sabrewing (*Campylopterus curvipennis*) [87], Azure-crowned Hummingbird (*Amazilia cyanocephala*) [38],[88], Broad-tailed Hummingbird (*Selasphorus platycercus*) [37] and Amethyst-throated Hummingbird (*Lampornis amethystinus*) [J. F. Ornelas, C. González, B. Hernández-Baños and J. García-Moreno, unpublished data]. Limited differentiation has been found in other species with a lowland distribution, such as the Rufous-tailed Hummingbird (*Amazilia tzacatl*) [89] and the Long-billed Hermit (*Phaethornis longirostris*) [90]. The Escudo Hummingbird (*A. t. handleyi*), endemic to the Caribbean island Escudo de Veraguas in western Panama, initially described as a distinct species on the basis of its considerably larger size and darker plumage, is slightly differentiated (ND2; 2 substitutions; uncorrected distance 0.2–0.5%) from the mainland *A. tzacatl* c. 10 km away [89].

Overall, population differentiation in the Mexican Sheartail seems primarily enhanced by isolation, which is reasonable for populations separated by a long distance. The Veracruz colonization hypothesis is consistent with the lower migration rate of the Veracruz population to Yucatan than was found for the opposite direction, and with the results of the tests of niche conservatism that suggest that the Veracruz colonization with gene flow was facilitated by niche similarity (PC1). Consequently, following geographic isolation, the populations of *D. eliza* separated by the Gulf of Mexico would have been exposed and eventually adapted to the different environmental conditions. Populations of the Mexican Sheartail separated by 780 km (and by the Gulf) are distributed in a unique environmental space, implying that the different environmental conditions in the Yucatan Peninsula and in Veracruz would have reduced gene flow, as shown by the IMa results, and this would have reinforced the divergence of the two mtDNA haplogroups following the initial spatial separation. This scenario is supported by our tests of niche divergence and conservatism that compared the amount of climatic divergence to the null expectation of background climatic divergence and that showed evidence for niche divergence between the *D. eliza* records of Veracruz and the Yucatan on two axes of environmental space related to annual precipitation and mean diurnal temperature range (PC2 and PC3). These findings support the hypothesis that climatic niche dissimilarity between *D. eliza* populations separated by the Gulf seems to have reduced gene flow. Our analyses of *D. eliza*, combining a phylogeographic and species distribution modeling approach, suggest that the observed patterns of genetic variation and divergence between the Veracruz and Yucatan populations are best explained by a combination of isolation exacerbated by subsequent climate differentiation between regions. Although the latter may be true for species that disperse poorly or that are reluctant to cross areas of less hospitable habitat for physiological reasons, niche divergence for other species with poor dispersal may mean enhanced opportunities for isolation and reduced gene flow, thereby increasing the likelihood of speciation.

Our morphological analysis confirmed that *D. eliza* hummingbirds from Veracruz are similar in most trait mean values to individuals from the Yucatan population (see also [12]). Patterns of limited population differentiation in size trait values were surprising given the large geographic separation between the two populations and habitat differences. Studies of hummingbirds, such as *A*. *melanogenys* [86], *C*. *curvipennis* [87], *A*. *cyanocephala* [38], and a member of the Mellisugini, *S*. *platycercus* [37], found significant size differences between populations in different habitats, yet separated by shorter distances. In all these cases, the genetic break at the potential barriers corresponds to differences in morphology and to the lack of overlap in environmental space between lineages on both sides of the barrier. One possible explanation for this pattern is that vicariance and ecological divergence have both played an important role in the strong morphological differentiation between populations that are physically separated [38],[86]–[87]. While this hypothesis may hold true for these hummingbird species, for which divergence times between populations were estimated to have occurred c. 700,000 years ago, the hypothesis alone is insufficient to explain the limited morphological variation between the Veracruz and Yucatan populations of *D. eliza*. Interestingly, the time of divergence between *D. eliza* populations was estimated at 120,000 years ago, supporting the hypothesis of a short period of isolation and limited morphological differentiation. However, male individuals from the Yucatan population had smaller values for the outermost rectrices than did males from Veracruz. It remains to be tested with larger sample sizes whether these differences represent significant levels of variation affecting the males’ acrobatic displays ([10], **Figure S1** and **Video S1**), and thus increased sexual selection in the smaller population of Veracruz.

### Conservation and Management Considerations

Our results reveal that the Veracruz and Yucatan populations of *D. eliza* are genetically differentiated, and that the outermost rectrices of male hummingbirds from Veracruz are longer than those of the males from Yucatan. The Mexican Sheartail Hummingbird is globally near threatened and both the Veracruz and Yucatan populations are locally endangered with population declines in Veracruz resulting from severe habitat degradation caused by livestock grazing, sugarcane cultivation and residential development, while the Yucatan population is under pressure mainly from the development of its coastal dune habitat for tourism [1],[13]–[15]. Here we have identified that the disjunct populations of *D. eliza* constitute distinct genetic lineages, and that the importance of these populations as reservoirs of endemic genetic diversity require different management approaches and merit targeted conservation efforts to preserve the unique genetic pools of both populations and their habitats.

## Supporting Information

Figure S1 **Stills from video recording, showing moments of a rocking pendulum flight displayed by a** ***Doricha eliza*** **male to a female at the nest.** (A) Photograph shows a male *D. eliza* from the Veracruz population. Photograph by Gerardo Sánchez Vigil. (B) Photograph shows a female *D. eliza* from the Veracruz population. Photograph by Yuyini Licona Vera. (C–J) The male begins the courtship display doing a pendulum flight (from left to right) in front of the female. During the display, the male extends his throat feathers and fully displays tail rectrices, while approaching the female repeatedly. The entire time, the female at the nest follows the male’s movements (red arrows). This pendulum flight is done repeatedly around the female (from right to left and from left to right) and is finished with an upward flight (not shown in the video). The video is available as supplementary material – Video S1.
(TIF)

Figure S2 **Bayesian posterior probabilities and bootstrap support for MrBayes and Maximum Likelihood analyses.** Illustration of tree topology based on ND2 sequences for North American and South American members of the Mellisugini clade. Values above branches denote posterior probabilities (PP) and those below branches denote bootstrap values.
(TIF)

Figure S3 **Bayesian posterior probabilities and bootstrap support for MrBayes and Maximum Likelihood analyses.** Illustration of tree topology based on the nuDNA locus 20454 unphased sequences from *D. eliza* and outgroups. Values above branches denote posterior probabilities (PP) and those below branches denote bootstrap values.
(TIF)

Figure S4 **Morphological differences between the Veracruz and Yucatan populations of** ***D. eliza*** **males.** Data are means and 95% confidence intervals for total body length (A), exposed culmen (B), base bill-width (C), wing chord (D), length of right outermost rectrix (E), and length of left outermost rectrix (F). Measurements are in mm.
(TIF)

Figure S5 **Morphological differences between the Veracruz and Yucatan populations of** ***D. eliza*** **females.** Data are means and 95% confidence intervals for total body length (A), exposed culmen (B), base bill-width (C), wing chord (D), and tail length (E). Measurements are in mm.
(TIF)

Table S1 **Code for identification (ID), sex, state and locality of origin, geographic coordinates and elevation of sampled individuals of** ***Doricha eliza*** **.**
(DOC)

Table S2 **Species names, sequence data and GenBank accession numbers for** ***Doricha eliza*** **(25) and outgroups (51) used in this study.**
(DOC)

Table S3 **Code for identification (ID), sex, morphological data and GenBank accession numbers for** ***Doricha eliza*** **individuals used in this study.**
(DOC)

Video S1 **Rocking pendulum flight as displayed by male Mexican Sheartails.** The video was recorded at Miradores, Veracruz during the breeding season (07 September 2012).
(MP4)
