# Supplementary figures and images for: Genetic, Ecological and Morphological Divergence between Populations of the Endangered Mexican Sheartail Hummingbird (Doricha eliza)
Source: PLoS One. 2014 Jul 3;9(7):e101870. doi: 10.1371/journal.pone.0101870 (PMC4081810; doi:10.1371/journal.pone.0101870)

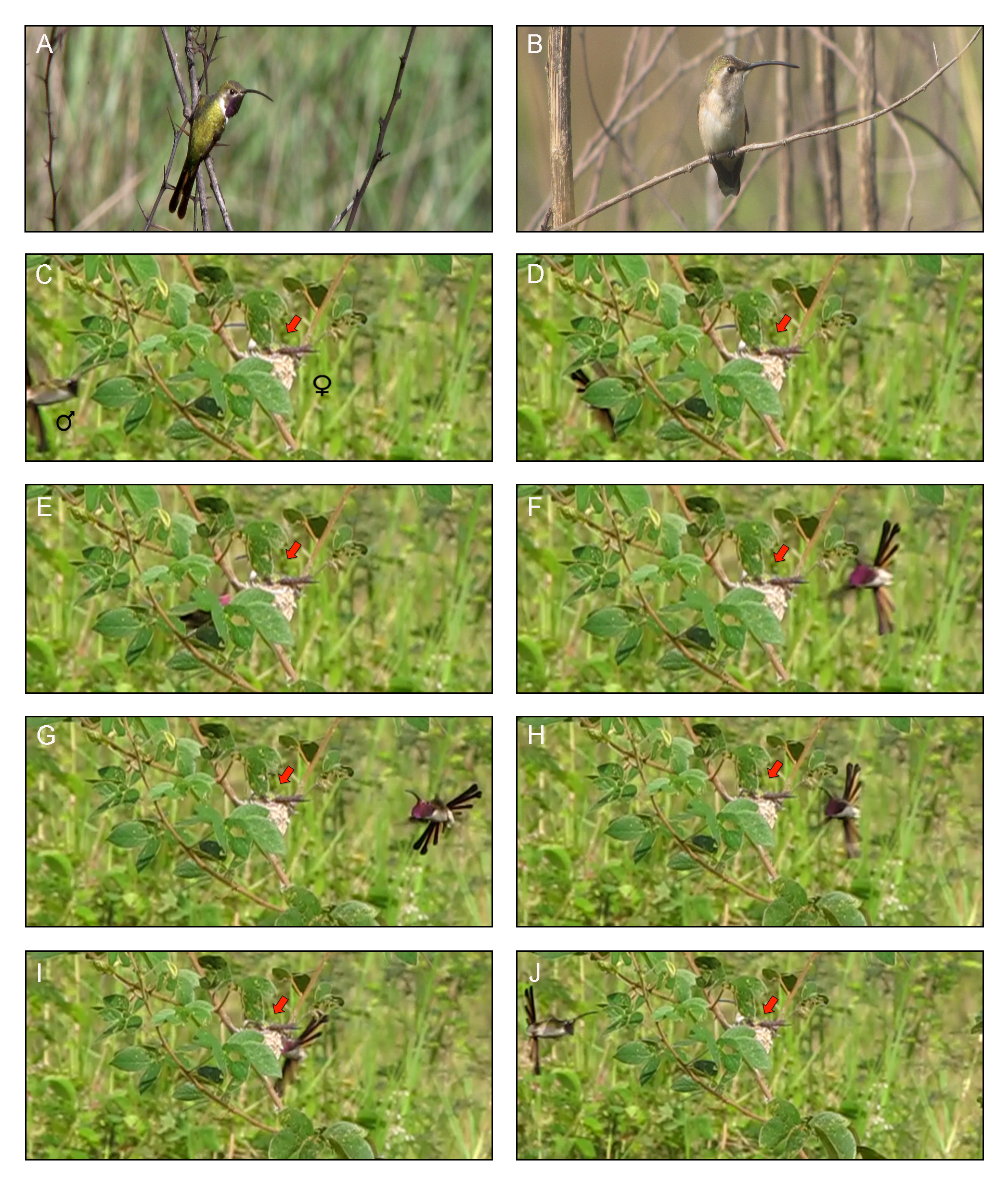

Supplement: Figure S1 — Stills from video recording, showing moments of a rocking pendulum flight displayed by a Doricha eliza male to a female at the nest. (A) Photograph shows a male D. eliza from the Veracruz population. Photograph by Gerardo Sánchez Vigil. (B) Photograph shows a female D. eliza from the Veracruz population. Photograph by Yuyini Licona Vera. (C–J) The male begins the courtship display doing a pendulum flight (from left to right) in front of the female. During the display, the male extends his throat feathers and fully displays tail rectrices, while approaching the female repeatedly. The entire time, the female at the nest follows the male’s movements (red arrows). This pendulum flight is done repeatedly around the female (from right to left and from left to right) and is finished with an upward flight (not shown in the video). The video is available as supplementary material – Video S1. (TIF) [file pone.0101870.s001.tif]

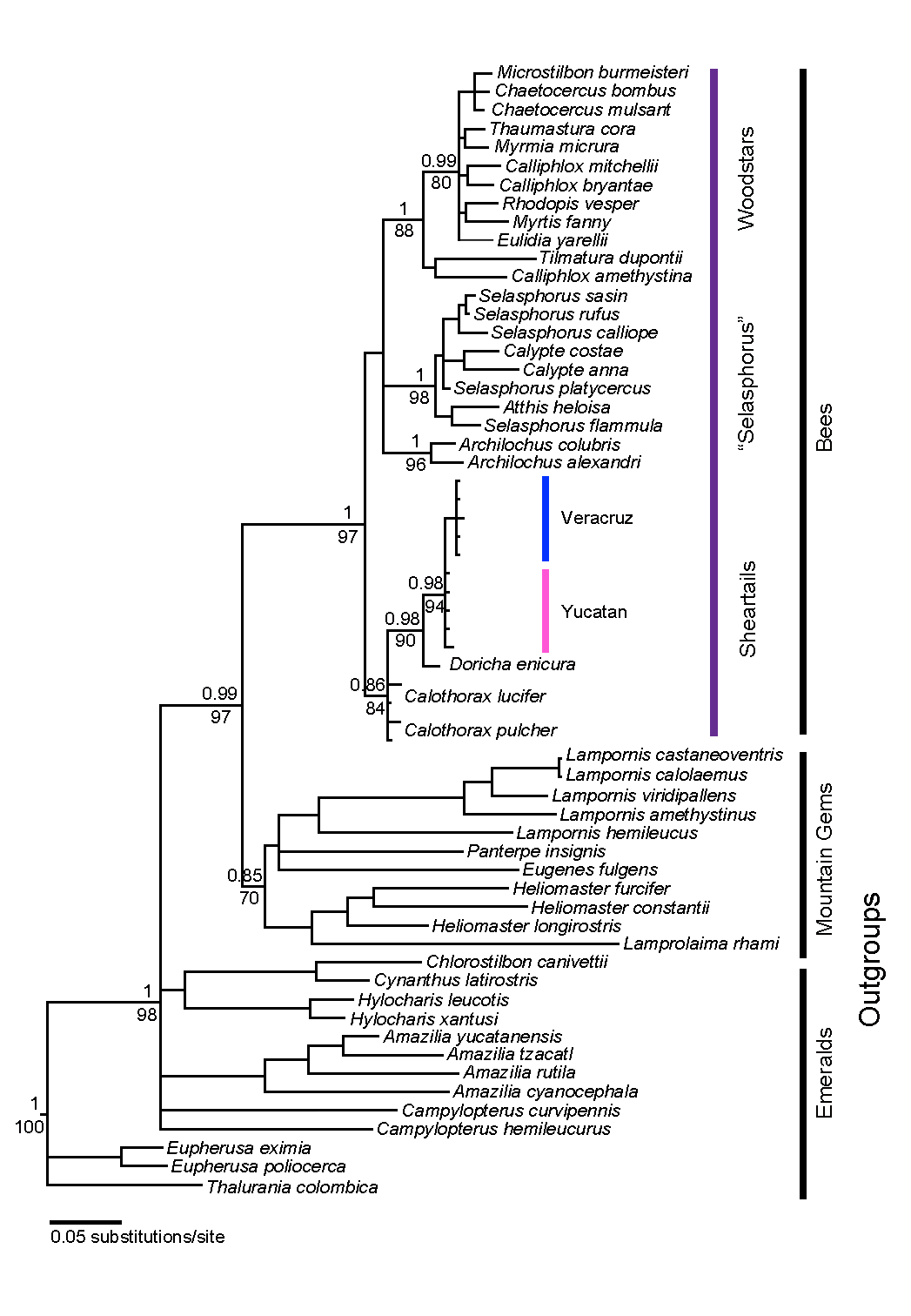

Supplement: Figure S2 — Bayesian posterior probabilities and bootstrap support for MrBayes and Maximum Likelihood analyses. Illustration of tree topology based on ND2 sequences for North American and South American members of the Mellisugini clade. Values above branches denote posterior probabilities (PP) and those below branches denote bootstrap values. (TIF) [file pone.0101870.s002.tif]

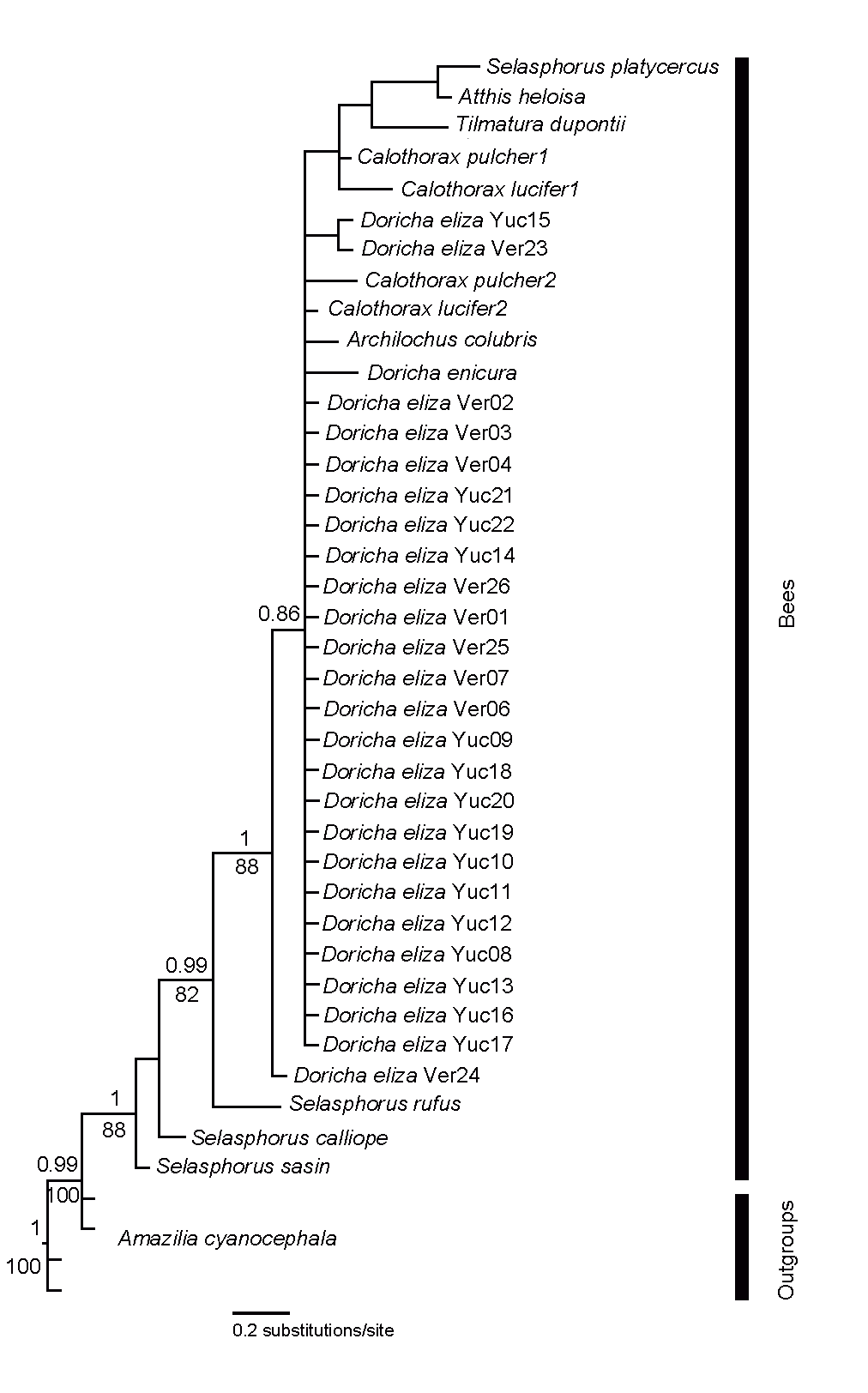

Supplement: Figure S3 — Bayesian posterior probabilities and bootstrap support for MrBayes and Maximum Likelihood analyses. Illustration of tree topology based on the nuDNA locus 20454 unphased sequences from D. eliza and outgroups. Values above branches denote posterior probabilities (PP) and those below branches denote bootstrap values. (TIF) [file pone.0101870.s003.tif]

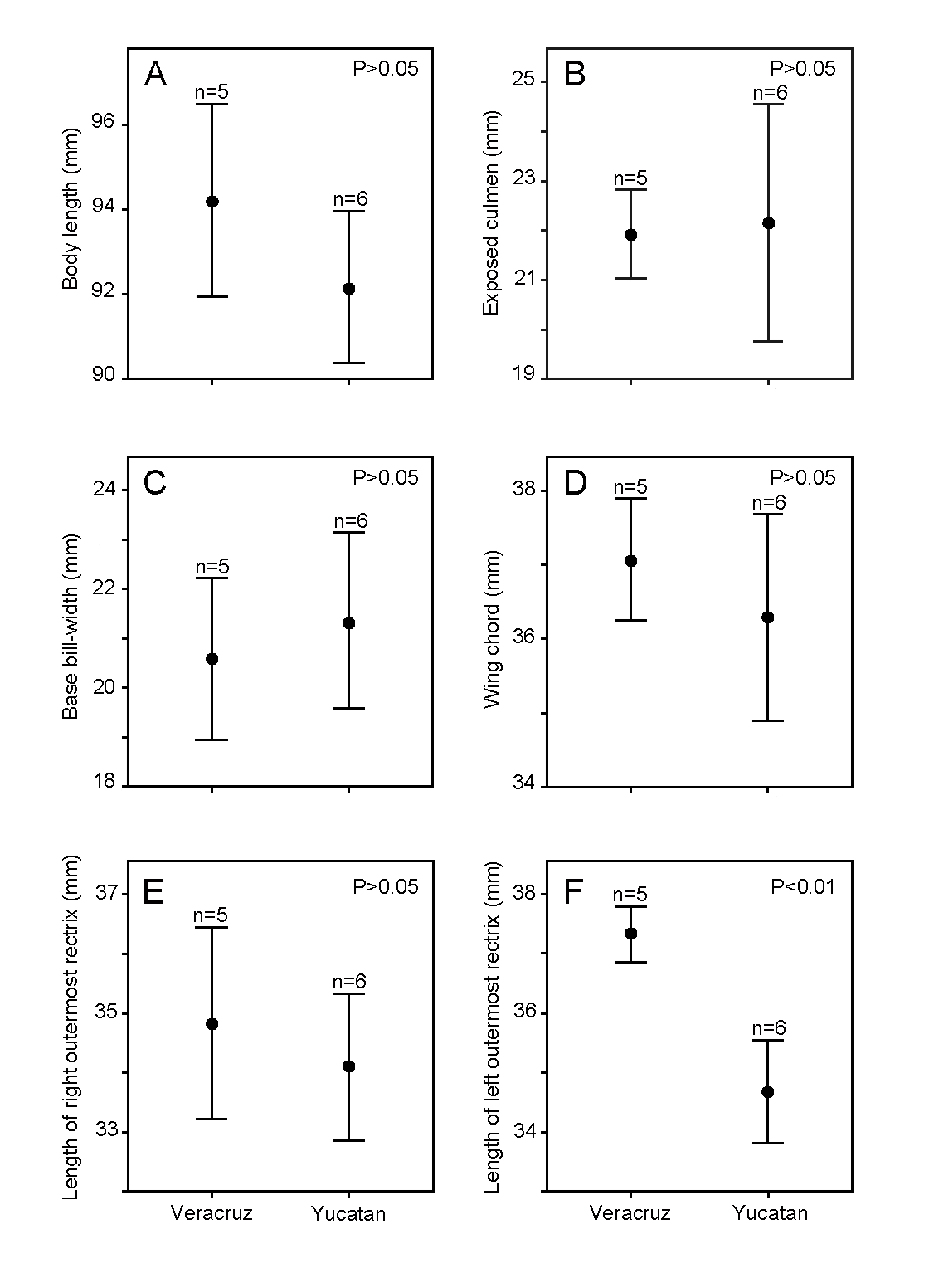

Supplement: Figure S4 — Morphological differences between the Veracruz and Yucatan populations of D. eliza males. Data are means and 95% confidence intervals for total body length (A), exposed culmen (B), base bill-width (C), wing chord (D), length of right outermost rectrix (E), and length of left outermost rectrix (F). Measurements are in mm. (TIF) [file pone.0101870.s004.tif]

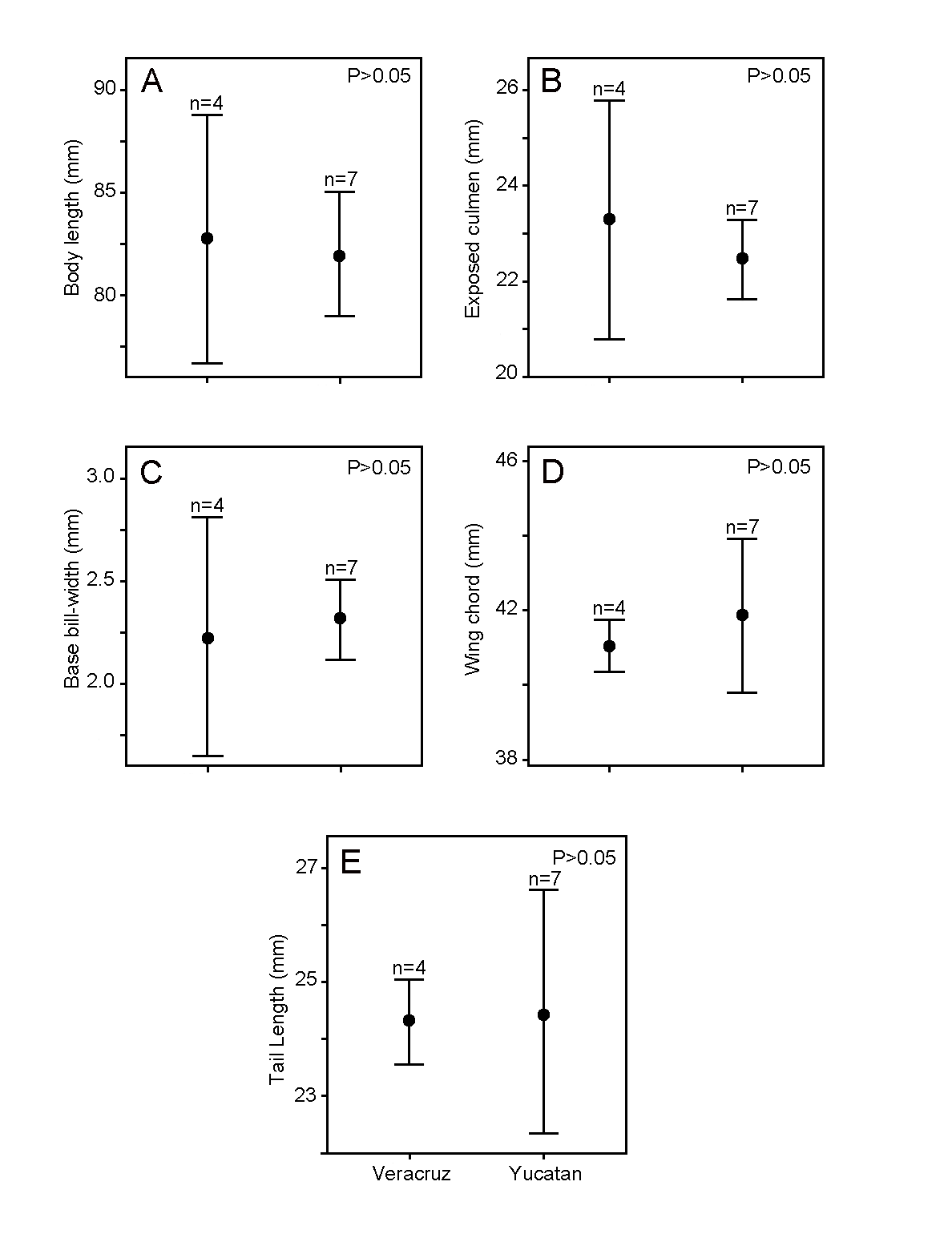

Supplement: Figure S5 — Morphological differences between the Veracruz and Yucatan populations of D. eliza females. Data are means and 95% confidence intervals for total body length (A), exposed culmen (B), base bill-width (C), wing chord (D), and tail length (E). Measurements are in mm. (TIF) [file pone.0101870.s005.tif]
