# Supplementary material for: Genetic, Ecological and Morphological Divergence between Populations of the Endangered Mexican Sheartail Hummingbird (Doricha eliza)
Source: PLoS One. 2014 Jul 3;9(7):e101870. doi: 10.1371/journal.pone.0101870 (PMC4081810; doi:10.1371/journal.pone.0101870)
Supplement: Table S1 — Code for identification (ID), sex, state and locality of origin, geographic coordinates and elevation of sampled individuals of Doricha eliza . (DOC) [file pone.0101870.s006.doc]

**Table S1. Code for identification (ID), sex, state and locality of origin, geographic coordinates and elevation of sampled individuals of *Doricha eliza*.**

|  |  |  |  |  |  |  |  |
| --- | --- | --- | --- | --- | --- | --- | --- |
| **N°** | **ID** | **Sex** | **State** | **Locality** | **Latitude**  **(N)** | **Longitude**  **(W)** | **Elevation**  **(m a.s.l.)** |
| 1 | VER01Len | F | Veracruz | El Lencero | 19°29´0.61´´ | 96°49´39.8´´ | 910 |
| 2 | VER02Len | F | Veracruz | El Lencero | 19°29´0.61´´ | 96°49´39.8´´ | 910 |
| 3 | VER03Mir | F | Veracruz | Miradores | 19°27´47.4´´ | 96°47´11.9´´ | 916 |
| 4 | VER04Xal | - | Veracruz | Jalapa | - | - | - |
| 5 | VER06Cha | F | Veracruz | Chavarrillo | 19°26´10´´ | 96°48´11.7´´ | 872 |
| 6 | VER07Len | M | Veracruz | El Lencero | 19°29´0.61´´ | 96°49'39.8´´ | 910 |
| 7 | VER23Len | M | Veracruz | El Lencero | 19°29´0.61´´ | 96°49´39.8´´ | 910 |
| 8 | VER24Len | M | Veracruz | El Lencero | 19°29´0.61´´ | 96°49´39.8´´ | 910 |
| 9 | VER25Len | M | Veracruz | El Lencero | 19°29´0.61´´ | 96°49´39.8´´ | 910 |
| 10 | VER26Act | M | Veracruz | Actopan | 19°33´6.00´´ | 96°22´41.0´´ | 20 |
| 11 | YUC08RLa | F | Yucatán | Río Lagartos | 21°35´17.6´´ | 88°09'14.2´´ | 9 |
| 12 | YUC09RLa | F | Yucatán | Río Lagartos | 21°34´55.9´´ | 88°09'24.5´´ | 12 |
| 13 | YUC10RLa | F | Yucatán | Río Lagartos | 21°34´55.9´´ | 88°09'24.5´´ | 12 |
| 14 | YUC11RLa | F | Yucatán | Río Lagartos | 21°34´55.9´´ | 88°09'24.5´´ | 12 |
| 15 | YUC12RLa | F | Yucatán | Río Lagartos | 21°35´17.6´´ | 88°09'14.2´´ | 9 |
| 16 | YUC13RLa | F | Yucatán | Río Lagartos | 21°35´17.6´´ | 88°09'14.2´´ | 9 |
| 17 | YUC14RLa | M | Yucatán | Río Lagartos | 21°34´55.9´´ | 88°09'24.5´´ | 12 |
| 18 | YUC15RLa | M | Yucatán | Río Lagartos | 21°34´55.9´´ | 88°09'24.5´´ | 12 |
| 19 | YUC16RLa | M | Yucatán | Río Lagartos | 21°34´55.9´´ | 88°09'24.5´´ | 12 |
| 20 | YUC17Chi | F | Yucatán | Chicxulub | 21°17´38.4´´ | 89°36'37.4´´ | 0 |
| 21 | YUC18Chi | M | Yucatán | Chicxulub | 21°17´38.4´´ | 89°36´37.4´´ | 0 |
| 22 | YUC19Chi | M | Yucatán | Chicxulub | 21°17´38.4´´ | 89°36´37.4´´ | 0 |
| 23 | YUC20Chi | M | Yucatán | Chicxulub | 21°17´38.4´´ | 89°36´37.4´´ | 0 |
| 24 | YUC21Chi | M | Yucatán | Chicxulub | 21°17´38.4´´ | 89°36´37.4´´ | 0 |
| 25 | YUC22Chi | M | Yucatán | Chicxulub | 21°17´38.4´´ | 89°36´37.4´´ | 0 |
