# Supplementary material for: Genetic, Ecological and Morphological Divergence between Populations of the Endangered Mexican Sheartail Hummingbird (Doricha eliza)
Source: PLoS One. 2014 Jul 3;9(7):e101870. doi: 10.1371/journal.pone.0101870 (PMC4081810; doi:10.1371/journal.pone.0101870)
Supplement: Table S2 — Species names, sequence data and GenBank accession numbers for Doricha eliza (25) and outgroups (51) used in this study. (DOC) [file pone.0101870.s007.doc]

**Table S2. Species names, sequence data and GenBank accession numbers for *Doricha eliza* (25) and outgroups (53) used in this study**.

|  |  |  |  | | |  | | | | | |
| --- | --- | --- | --- | --- | --- | --- | --- | --- | --- | --- | --- |
| **N^o^** | **Species** | | | **Institution or Museum** | **Voucher tag or other identifier** | |  | **GenBank Accession Numbers** | | |  |
|  |  | | |  |  | | ***ND2*** | | ***ATPase 6–8*** | ***20454*** | |
| 1 | *Amazilia cyanocephala* | | | INECOL | Ac-INE-1 | | KC858470 | | JX050079 | KJ710558 | |
| 2 | *Amazilia cyanocephala* | | | INECOL | Ac-Ord-1 | | KC858469 | | JX050062 | KJ710559 | |
| 3 | *Amazilia cyanocephala* | | | INECOL | Ac-Gua-1 | | KC858479 | | KF640798 | KJ710556 | |
| 4 | *Amazilia cyanocephala* | | | INECOL | Ac-Gua-2 | | KC858480 | | KF640804 | KJ710557 | |
| 5 | *Amazilia cyanocephala* | | | INECOL | VER 119 | | KC858467 | | - | - | |
| 6 | *Amazilia rutila* | | | INECOL | Ar-3 | | KC858498 | | - | - | |
| 7 | *Amazilia tzacatl* | | | UAM | CAM350 | | EU983306 | | - | - | |
| 8 | *Amazilia yucatanensis* | | | INECOL | Ay-HUIT8 | | KC858503 | | - | - | |
| 9 | *Archilochus alexandri* | | | LSUMZ | B-21848 | | EU042529 | | - | - | |
| 10 | *Archilochus colubris* | | | INECOL | ACO02Yuc | | KJ710597 | | KJ710519 | KJ710560 | |
| 11 | *Atthis heloisa* | | | INECOL | OMVP 1041 | | KC858534 | | KJ710520 | KJ710561 | |
| 12 | *Calliphlox amethystina* | | | NMNH | b-10703 | | GU167211 | | - | - | |
| 13 | *Calliphlox bryantae* | | | LSUMNS | B-28180 | | EU042531 | | - | - | |
| 14 | *Calliphlox mitchellii* | | | LSUMNS | B-12194 | | AY830516 | | - | - | |
| 15 | *Calothorax lucifer1* | | | INECOL | DF136 | | KC858411 | | KJ710521 | KJ710562 | |
| 16 | *Calothorax lucifer2* | | | INECOL | CAL02 | | KJ710598 | | KJ710522 | KJ710563 | |
| 17 | *Calothorax pulcher1* | | | INECOL | PUE135 | | KC858531 | | KJ710524 | KJ710565 | |
| 18 | *Calothorax pulcher2* | | | INECOL | OAX02 | | KJ710599 | | KJ710523 | KJ710564 | |
| 19 | *Calypte anna* | | | LSUMNS | B-24864 | | EU042532 | | - | - | |
| 20 | *Calypte costae* | | | LSUMNS | B-21595 | | EU042533 | | - | - | |
| 21 | *Campylopterus curvipennis* | | | MZFC | CHIMA 080 | | KC858426 | | - | - | |
| 22 | *Campylopterus hemileucurus* | | | INECOL | CHIS 113 | | EU042534 | | - | - | |
| 23 | *Chaetocercus bombus* | | | LSUMNS | B-5225 | | GU167213 | | - | - | |
| 24 | *Chaetocercus mulsant* | | | LSUMNS | B-6301 | | AY830456 | | - | - | |
| 25 | *Chlorostilbon canivettii* | | | MZFC | BEHB 060 | | KC858433 | | - | - | |
| 26 | *Cynanthus latirostris* | | | MZFC | FJSS 053 | | KC858435 | | - | - | |
| 27 | *Doricha eliza* | | | INECOL | VER01Len | | KJ710601 | | KJ710525 | KJ710566 | |
| 28 | *Doricha eliza* | | | INECOL | VER02Len | | KJ710602 | | KJ710526 | KJ710567 | |
| 29 | *Doricha eliza* | | | INECOL | VER03Mir | | KJ710603 | | KJ710527 | KJ710568 | |
| 30 | *Doricha eliza* | | | INECOL | VER04Xal | | KC858530 | | KJ710528 | KJ710569 | |
| 31 | *Doricha eliza* | | | INECOL | VER06Cha | | KJ710604 | | KJ710529 | KJ710570 | |
| 32 | *Doricha eliza* | | | INECOL | VER07Len | | KJ710605 | | KJ710530 | KJ710571 | |
| 33 | *Doricha eliza* | | | INECOL | YUC08RLa | | KJ710610 | | KJ710535 | KJ710576 | |
| 34 | *Doricha eliza* | | | INECOL | YUC09RLa | | KJ710611 | | KJ710536 | KJ710577 | |
| 35 | *Doricha eliza* | | | INECOL | YUC10RLa | | KJ710612 | | KJ710537 | KJ710578 | |
| 36 | *Doricha eliza* | | | INECOL | YUC11RLa | | KJ710613 | | KJ710538 | KJ710579 | |
| 37 | *Doricha eliza* | | | INECOL | YUC12RLa | | KJ710614 | | KJ710539 | KJ710580 | |
| 38 | *Doricha eliza* | | | INECOL | YUC13RLa | | KJ710615 | | KJ710540 | KJ710581 | |
| 39 | *Doricha eliza* | | | INECOL | YUC14RLa | | KJ710616 | | KJ710541 | KJ710582 | |
| 40 | *Doricha eliza* | | | INECOL | YUC15RLa | | KJ710617 | | KJ710542 | KJ710583 | |
| 41 | *Doricha eliza* | | | INECOL | YUC16RLa | | KJ710618 | | KJ710543 | KJ710584 | |
| 42 | *Doricha eliza* | | | INECOL | YUC17Chi | | KJ710619 | | KJ710544 | KJ710585 | |
| 43 | *Doricha eliza* | | | INECOL | YUC18Chi | | KJ710620 | | KJ710545 | KJ710586 | |
| 44 | *Doricha eliza* | | | INECOL | YUC19Chi | | KJ710621 | | KJ710546 | KJ710587 | |
| 45 | *Doricha eliza* | | | INECOL | YUC20Chi | | KJ710622 | | KJ710547 | KJ710588 | |
| 46 | *Doricha eliza* | | | INECOL | YUC21Chi | | KJ710623 | | KJ710548 | KJ710589 | |
| 47 | *Doricha eliza* | | | INECOL | YUC22Chi | | KJ710624 | | KJ710549 | KJ710590 | |
| 48 | *Doricha eliza* | | | INECOL | VER23Len | | KJ710606 | | KJ710531 | KJ710572 | |
| 49 | *Doricha eliza* | | | INECOL | VER24Len | | KJ710607 | | KJ710532 | KJ710573 | |
| 50 | *Doricha eliza* | | | INECOL | VER25Len | | KJ710608 | | KJ710533 | KJ710574 | |
| 51 | *Doricha eliza* | | | INECOL | VER26Act | | KJ710609 | | KJ710534 | KJ710575 | |
| 52 | *Doricha enicura* | | | INECOL | DEN14Com | | KJ710600 | | KJ710550 | KJ710591 | |
| 53 | *Eugenes fulgens* | | | INECOL | CHIS 142 | | AY830481 | | - | - | |
| 54 | *Eulidia yarellii* | | | WF | WV 039 | | JQ025412 | | - | - | |
| 55 | *Eupherusa eximia* | | | MZFC | CHIMA 146 | | EU042552 | | - | - | |
| 56 | *Eupherusa poliocerca* | | | MZFC | OMVP 655 | | KC858522 | | - | - | |
| 57 | *Heliomaster constantii* | | | MZFC | OMVP 750 | | KC858529 | | - | - | |
| 58 | *Heliomaster furcifer* | | | LSUMNS | B-6709 | | GU167234 | | - | - | |
| 59 | *Heliomaster longirostris* | | | INECOL | - | | KC858528 | | - | - | |
| 60 | *Hylocharis leucotis* | | | MZFC | OMVP 304 | | KC858440 | | - | - | |
| 61 | *Hylocharis xantusi* | | | MZFC | - | | EU543353 | | - | - | |
| 62 | *Lampornis amethystinus* | | | MZFC | BMM057 | | EU543341 | | - | - | |
| 63 | *Lampornis calolaemus* | | | LSUMZ | B-28169 | | EU042565 | | - | - | |
| 64 | *Lampornis castaneoventris* | | | LSUMNS | B-28257 | | EU042566 | | - | - | |
| 65 | *Lampornis hemileucus* | | | LSUMNS | B-16006 | | EU042567 | | - | - | |
| 66 | *Lampornis viridipallens* | | | MZFC | - | | EU543355 | | - | - | |
| 67 | *Lamprolaima rhami* | | | INECOL | Lr-5 | | KC858527 | | - | - | |
| 68 | *Microstilbon burmeisteri* | | | ZMC | 114832 | | GU167242 | | - | - | |
| 69 | *Myrmia micrura* | | | LSUMNS | B-5233 | | GU167243 | | - | - | |
| 70 | *Myrtis fanny* | | | LSUMNS | B-3592 | | AY830503 | | - | - | |
| 71 | *Panterpe insignis* | | | LSUMNS | B-16264 | | AY830509 | | - | - | |
| 72 | *Rhodopis vesper* | | | LSUMNS | B-14277 | | EU042588 | | - | - | |
| 71 | *Selasphorus flammula* | | | LSUMNS | B-28269 | | EU042589 | | - | - | |
| 72 | *Selasphorus platycercus* | | | INECOL | SWRS108 | | KF792850 | | KJ710552 | KJ710593 | |
| 73 | *Selasphorus rufus* | | | INECOL | BMM475 | | EU042590 | | KJ710553 | KJ710594 | |
| 74 | *Selasphorus sasin* | | | INECOL | SELSASI | | KF792865 | | KJ710554 | KJ710595 | |
| 75 | *Selasphorus calliope* | | | INECOL | STECALL | | KF792866 | | KJ710551 | KJ710592 | |
| 76 | *Thalurania colombica* | | | LSUMNS | B-11793 | | AY830524 | | - | - | |
| 77 | *Thaumastura cora* | | | MSB | Bird33004 | | JQ025424 | | - | - | |
| 78 | *Tilmatura dupontii* | | | INECOL | Pd-1 | | KC858535 | | KJ710555 | KJ710596 | |

### Museum abbreviations used in voucher numbers: INECOL = Instituto de Ecología, AC, Xalapa, Veracruz, México; LSUMNS = Collection of Genetic Resources, Louisiana State University Museum of Natural Science; NMNH = National Museum of Natural History, MSB = Museum of Southwestern Biology, WV = West Virginia Museum, MZFC = Museo de Zoología de la Facultad de Ciencias, ZMC = Zoologist Museum of Copenhagen.
