# Supplementary material for: Genetic, Ecological and Morphological Divergence between Populations of the Endangered Mexican Sheartail Hummingbird (Doricha eliza)
Source: PLoS One. 2014 Jul 3;9(7):e101870. doi: 10.1371/journal.pone.0101870 (PMC4081810; doi:10.1371/journal.pone.0101870)
Supplement: Table S3 — Code for identification (ID), sex, morphological data and GenBank accession numbers for Doricha eliza individuals used in this study. (DOC) [file pone.0101870.s008.doc]

**Table S3. Code for identification (ID), sex, morphological data and GenBank accession numbers of sampled individuals of *Doricha eliza.***

|  |  | |  | |  |  |  |  | |  | |  | |  |  | | |
| --- | --- | --- | --- | --- | --- | --- | --- | --- | --- | --- | --- | --- | --- | --- | --- | --- | --- |
| **ID** | **Sex** | **Total**  **length (mm)** | | **Exposed**  **culmen (mm)** | **Bill**  **width (mm)** | **Wing**  **chord (mm)** | **Tail**  **length (mm)** | | **r5**  **left**  **(mm)** | | **r5**  **right**  **(mm)** | | **GenBank Accession Numbers** | | | | |
|  |  |  |  |  |  |  |  |  |  |  |  |  | ***ND2*** | | | ***ATPase 6–8*** | ***20454*** |
|  |  |  | |  |  |  |  | |  | |  | |  | | |  |  |
| VER01Len | F | 75 | | 19.5 | 1.8 | 40.0 | 24.4 | | ND | | ND | | KJ710601 | | | KJ710525 | KJ710566 |
| VER02Len | F | 82 | | 24.5 | 2.7 | 41.2 | 23.0 | | ND | | ND | | KJ710602 | | | KJ710526 | KJ710567 |
| VER03Mir | F | 90 | | 24.6 | 1.6 | 41.6 | 24.3 | | ND | | ND | | KJ710603 | | | KJ710527 | KJ710568 |
| VER04Xal | ND | ND | | ND | ND | ND | ND | | ND | | ND | | KC858530 | | | KJ710528 | KJ710569 |
| VER06Cha | F | 84 | | 24.6 | 2.8 | 41.3 | 24.4 | | ND | | ND | | KJ710604 | | | KJ710529 | KJ710570 |
| VER07Len | M | 98 | | 20.7 | 1.8 | 35.7 | ND | | 35.8 | | 36.7 | | KJ710605 | | | KJ710530 | KJ710571 |
| YUC08RLa | F | 77 | | 23.0 | 2.4 | 42.2 | 23.9 | | ND | | ND | | KJ710610 | | | KJ710535 | KJ710576 |
| YUC09RLa | F | 89 | | 22.2 | 2.0 | 41.5 | 28.8 | | ND | | ND | | KJ710611 | | | KJ710536 | KJ710577 |
| YUC10RLa | F | 80 | | 22.4 | 2.0 | 41.0 | 24.1 | | ND | | ND | | KJ710612 | | | KJ710537 | KJ710578 |
| YUC11RLa | F | 86 | | 22.1 | 2.2 | 44.2 | 26.7 | | ND | | ND | | KJ710613 | | | KJ710538 | KJ710579 |
| YUC12RLa | F | 81 | | 23.9 | 2.8 | 45.9 | 25.5 | | ND | | ND | | KJ710614 | | | KJ710539 | KJ710580 |
| YUC13RLa | F | 81 | | 23.3 | 2.4 | 41.1 | 22.3 | | ND | | ND | | KJ710615 | | | KJ710540 | KJ710581 |
| YUC14RLa | M^*^ | 75 | | 21.5 | 2.1 | 38.3 | ND | | 21.5 | | 22.0 | | KJ710616 | | | KJ710541 | KJ710582 |
| YUC15RLa | M | 91 | | 21.7 | 2.0 | 38.3 | ND | | 33.1 | | 33.7 | | KJ710617 | | | KJ710542 | KJ710583 |
| YUC16RLa | M | 93 | | 20.7 | 1.8 | 35.4 | ND | | 34.9 | | 34.2 | | KJ710618 | | | KJ710543 | KJ710584 |
| YUC17Chi | F | 80 | | 20.4 | 2.4 | 24.0 | 20.1 | | ND | | ND | | KJ710619 | | | KJ710544 | KJ710585 |
| YUC18Chi | M | 91 | | 28.2 | 2.2 | 36.5 | ND | | 31.5 | | 33.4 | | KJ710620 | | | KJ710545 | KJ710586 |
| YUC19Chi | M^*^ | 79 | | 22.3 | 2.0 | 37.9 | ND | | 25.6 | | 26.5 | | KJ710621 | | | KJ710546 | KJ710587 |
| YUC20Chi | M | 89 | | 20.5 | 2.1 | 33.3 | ND | | 34.8 | | 35.9 | | KJ710622 | | | KJ710547 | KJ710588 |
| YUC21Chi | M | 95 | | 20.6 | 2.5 | 36.7 | ND | | 35.8 | | 35.0 | | KJ710623 | | | KJ710548 | KJ710589 |
| YUC22Chi | M | 94 | | 21.1 | 2.2 | 37.4 | ND | | 34.5 | | 35.9 | | KJ710624 | | | KJ710540 | KJ710590 |
| VER23Len | M | 93 | | 22.1 | 2.1 | 36.5 | ND | | 34.7 | | 38.0 | | KJ710606 | | | KJ710531 | KJ710572 |
| VER24Len | M | 91 | | 22.8 | 2.1 | 37.2 | ND | | 34.7 | | 37.8 | | KJ710607 | | | KJ710532 | KJ710573 |
| VER25Len | M | 95 | | 21.1 | 2.0 | 37.8 | ND | | 37.0 | | 37.2 | | KJ710608 | | | KJ710533 | KJ710574 |
| VER26Act | M | 94 | | 23.0 | 2.3 | 38.0 | ND | | 32.0 | | 37.0 | | KJ710609 | | | KJ710534 | KJ710575 |

^*^ These male individuals were juveniles and were excluded from morphological analysis. r5 = outermost rectrix. ND = No data.
